# A Smart Mobile Health Tool Versus a Paper Action Plan to Support Self-Management of Chronic Obstructive Pulmonary Disease Exacerbations: Randomized Controlled Trial

**DOI:** 10.2196/14408

**Published:** 2019-10-09

**Authors:** Lonneke Boer, Erik Bischoff, Maarten van der Heijden, Peter Lucas, Reinier Akkermans, Jan Vercoulen, Yvonne Heijdra, Willem Assendelft, Tjard Schermer

**Affiliations:** 1 Department of Primary and Community Care Radboud Institute for Health Sciences Radboud University Medical Center Nijmegen Netherlands; 2 Institute for Computing and Information Science Radboud University Nijmegen Netherlands; 3 Department of Medical Psychology Radboud Institute for Health Sciences Radboud University Medical Center Nijmegen Netherlands; 4 Department of Pulmonary Diseases Radboud Institute for Health Sciences Radboud University Medical Center Nijmegen Netherlands; 5 Netherlands Institute for Health Services Research (NIVEL) Utrecht Netherlands

**Keywords:** COPD, symptom flare up, mHealth, self-management

## Abstract

**Background:**

Many patients with chronic obstructive pulmonary disease (COPD) suffer from exacerbations, a worsening of their respiratory symptoms that warrants medical treatment. Exacerbations are often poorly recognized or managed by patients, leading to increased disease burden and health care costs.

**Objective:**

This study aimed to examine the effects of a smart mobile health (mHealth) tool that supports COPD patients in the self-management of exacerbations by providing predictions of early exacerbation onset and timely treatment advice without the interference of health care professionals.

**Methods:**

In a multicenter, 2-arm randomized controlled trial with 12-months follow-up, patients with COPD used the smart mHealth tool (intervention group) or a paper action plan (control group) when they experienced worsening of respiratory symptoms. For our primary outcome exacerbation-free time, expressed as weeks without exacerbation, we used an automated telephone questionnaire system to measure weekly respiratory symptoms and treatment actions. Secondary outcomes were health status, self-efficacy, self-management behavior, health care utilization, and usability. For our analyses, we used negative binomial regression, multilevel logistic regression, and generalized estimating equation regression models.

**Results:**

Of the 87 patients with COPD recruited from primary and secondary care centers, 43 were randomized to the intervention group. We found no statistically significant differences between the intervention group and the control group in exacerbation-free weeks (mean 30.6, SD 13.3 vs mean 28.0, SD 14.8 weeks, respectively; rate ratio 1.21; 95% CI 0.77-1.91) or in health status, self-efficacy, self-management behavior, and health care utilization. Patients using the mHealth tool valued it as a more supportive tool than patients using the paper action plan. Patients considered the usability of the mHealth tool as good.

**Conclusions:**

This study did not show beneficial effects of a smart mHealth tool on exacerbation-free time, health status, self-efficacy, self-management behavior, and health care utilization in patients with COPD compared with the use of a paper action plan. Participants were positive about the supportive function and the usability of the mHealth tool. mHealth may be a valuable alternative for COPD patients who prefer a digital tool instead of a paper action plan.

**Trial Registration:**

ClinicalTrials.gov NCT02553096; https://clinicaltrials.gov/ct2/show/NCT02553096.

## Introduction

Exacerbations in chronic obstructive pulmonary disease (COPD) are acute events of transient worsening of the respiratory condition. Exacerbations considerably affect patients’ health status [1,2], accelerate the decline in lung function [3], and contribute to COPD-related costs [4]. Despite the substantial impact that exacerbations may have, patients with COPD often have difficulty in recognizing symptom deterioration [5] and do not respond timely or adequately in the course of symptom worsening [6].

Self-management strategies, such as the use of a written exacerbation action plan, have been shown to improve exacerbation outcomes, that is, decrease exacerbation duration [7,8], reduce hospital admissions [9,10], and decrease the impact on health status [10,11]. However, many patients do not adhere to the self-management instructions in their action plans when an exacerbation is imminent [7,12] and thus do not get the benefit of the favorable health effects of timely detection and subsequent intervention.

Telemonitoring, in which patients record and send information on symptoms or physiological measurements to a supervising clinician, may be an alternative approach to self-management strategies to reduce the impact of COPD exacerbations. Beneficial effects have been reported on the number of hospital admissions [13], emergency visits [13], and quality of life in some studies [14,15] but not in all [16]. There is much heterogeneity between telemonitoring interventions regarding devices and clinical content, and the amount of additional support that patients receive. However, in contrast with self-management, telemonitoring strongly depends on the judgement of the clinician and the patient is not expected to interpret his or her own symptoms and signs. Therefore, we have developed [17] and validated [18] an innovative mobile health (mHealth) tool, called the Adaptive Computerized COPD Exacerbation Self-management Support system. This tool aims to tailor self-management support more efficiently and continuously than with a written action plan but without heavily increasing the involvement of health care professionals to monitor input, as is the case with telemonitoring. The mHealth tool integrates information on symptom changes and physiological measurements (ie, pulse oximetry, spirometry, and measurement of body temperature) in an easy-to-use app by means of a mobile phone [17]. On the basis of a decision tree built by a clinical expert panel and a Bayesian prediction model, the tool provides automated, tailored self-management advice to the patient without the involvement of a health care professional [18]. Patients can use the tool at their own initiative to monitor symptom changes at any time of day or night and receive ad hoc, tailored advice.

In this study, we examined the clinical effectiveness of the mHealth tool. We hypothesized that in patients with COPD, the use of the tool would lead to more weeks without exacerbations; improvement in health status, self-efficacy, and self-management behavior; and a reduction in health care utilization compared with the use of a paper exacerbation action plan. We also evaluated how patients valued the tool’s supportive function and usability.

## Methods

### Study Design

This study was a multicenter, parallel, 2-arm, randomized controlled trial with a follow-up of 12 months per patient. After signing informed consent, patients with COPD recruited from general practices and outpatient clinics followed a 20-min self-management educational group session primarily addressing early recognition and prompt treatment of exacerbations. Subsequently, patients were randomized to either (1) usual care according to current COPD guidelines, that is, exacerbation self-management support through the use of a paper exacerbation action plan (control group) or (2) exacerbation self-management support through the use of the mHealth tool (intervention group). Participants in the control group were provided with a written action plan if they did not have one. Participants in the intervention group were instructed not to use their action plan if they had one before participation. This study has been registered at ClinicalTrials.gov (Identifier: NCT02553096) and has been approved by the medical ethics review board, region Arnhem-Nijmegen, the Netherlands (file 2014-1270).

### Setting and Participants

Patients were recruited between June 2015 and July 2016 at the pulmonary outpatient clinics of 3 Dutch hospitals and 9 general practices in the city of Nijmegen and the surroundings in the Netherlands. All participating centers delivered care according to the current Dutch COPD guideline [19] and had a pulmonary or practice nurse available for COPD care. Patients were eligible for participation if they (1) were aged at least 40 years, (2) had a spirometry-confirmed diagnosis of COPD (postbronchodilator forced expiratory volume in 1 second (FEV_1_)/forced vital capacity<0.7), and (3) had experienced 2 or more symptom-based exacerbations in the previous 12 months, defined as a change for greater than or equal to 2 consecutive days in either greater than or equal to 2 major symptoms (dyspnea, sputum purulence, and sputum amount) or any 1 major symptom plus greater than or equal to 1 minor symptoms (colds, wheeze, sore throat, and cough) [3,20]. Exclusion criteria were (1) severe comorbid conditions that prohibited safe participation, (2) insufficient knowledge of the Dutch language, and (3) persisting difficulties in using the mHealth system after a 2-week practice period and additional assistance.

### Randomization Procedure

We used a computer-generated 2-block randomization procedure, stratifying for the health care center. All patients from the participating centers who met the inclusion criteria received a questionnaire from their health care professional with questions related to exacerbations in the previous 12 months. Patients who responded and had experienced 2 or more symptom-based exacerbations in the previous 12 months (see inclusion criteria) were invited by the research team to participate in this study. The allocation order was determined by the order in which eligible patients responded to our invitation to participate (kept by the research assistant). Participants were assigned to one of the groups after signing informed consent during the group meeting by the researcher (LB). Owing to the type of intervention, patients and health care professionals could not be blinded for group assignment. In addition, the research team could not be blinded as it was responsible for the personalization and technical support of the mHealth tool. The study statistician (RA) who was responsible for analyzing the data was blinded for study assignment of the participants until the analyses had been finished.

### Intervention and Control Group

Before randomization and after signing the written informed consent, all participants received a 20-min educational session based on the Dutch version of the Living Well with COPD self-management program provided by the nurse in groups of 4 to 10 participants to establish a homogeneous baseline in exacerbation self-management knowledge [12].

Participants in the intervention group were instructed to visit the nurse within 2 weeks after allocation for instructions on the use of the mHealth tool. The tool consisted of a mobile phone (provided by the research team), a pulse oximeter (CMS50D, Contec Medical Systems,), a spirometer (PiKo-1 monitor, nSpire), and a forehead thermometer (FTN, Medisana AG). Patients answered 12 yes-or-no questions concerning changes in symptoms, physical limitations, and emotions using the touch screen on the mobile phone complemented by measurements with the pulse oximeter, spirometer, and forehead thermometer (see Multimedia Appendix 1) [17]. All questions had to be answered to proceed. On the basis of a built-in Bayesian network decision model, the mHealth tool then provided one or more of the following advices: (1) increase your bronchodilator use (including a personalized medication instruction), (2) use your breathing techniques, (3) use your coughing techniques, (4) be thoughtful of how you distribute your energy during the day, (4) contact your health care professional today, (5) measure again tomorrow. Completing the questions and measurements took approximately 5 min. The mHealth tool has been developed in close collaboration with COPD patients and health care professionals [17] and has shown high sensitivity and specificity [18].

Before the trial started, participants in the intervention group were instructed to use the system daily for 2 weeks to get familiarized with the app, mobile phone, spirometer, pulse oximeter, and forehead thermometer. Data were sent to a secured Web-based interface and were monitored by the research team to make sure participants practiced sufficiently. After this 2-week run-in period, the nurses evaluated patients’ use of the system, including the physiological measurements. Reference values for each patient’s FEV_1_ and peripheral oxygen saturation were set. Then, the 12-month follow-up period started. Patients were instructed to use the tool every time they experienced or had any doubts about any change in symptoms or disease burden.

Participants in the control group visited the nurse within 2 weeks of allocation for instructions on the use of a paper exacerbation action plan. When patients did not already possess a written action plan at that moment, the nurses provided the action plan of the Living Well with COPD program [12]. The plan consisted of instructions regarding the self-management of an exacerbation, for example, increase the use of bronchodilators, initiate a standing prescription for a course of prednisolone and/or antibiotic treatment if applicable, or contact the health care professional within 3 days of symptom aggravation.

At the 3-month follow-up, patients in both the intervention and control groups were invited by their nurse to evaluate their self-management of COPD exacerbations. In the intervention group, only the nurses received the patients’ entries in the mHealth tool from the research team to enable tailoring of feedback on self-management behavior. In the control group, the nurses evaluated the use of the paper action plan. Patients in both the groups did not receive any feedback on self-management behavior before or after this nurse contact. All patients in both the intervention and control groups continued to have complete access to their health care professionals during the follow-up.

### Outcomes and Follow-Up

Our primary outcome was the difference in the number of exacerbation-free weeks between the intervention and control groups. An exacerbation-free week was defined as a week in which there had not been episodes of 2 or more consecutive days with worsening of 2 major symptoms (ie, dyspnea, sputum purulence, and sputum amount) or 1 major and 1 or more minor symptoms (ie, colds, wheeze, sore throat, and cough) [21]. Symptom changes were assessed using the Telephonic Exacerbation Assessment System (TEXAS, Radboudumc), an automated telephone call system that contacted participants weekly on the day and time of their preference [22]. TEXAS consisted of closed questions regarding changes in respiratory symptoms, use of health care resources, and use of respiratory medication in the week before the call, and its validity has been demonstrated previously [22]. Owing to the discontinuation of the contract with the provider of TEXAS, the last 19 participants in the trial received a weekly online questionnaire containing the same questions as TEXAS. These participants used both measuring tools during 2 weeks before stopping with TEXAS, which enabled us to compare data entries from TEXAS with the online survey tool. We found no differences in the data entries.

The secondary outcomes included the following:

- Exacerbation-related outcomes, that is, the number of unscheduled health care contacts, the number of exacerbations treated with antibiotics and/or prednisolone, and the number of exacerbation-related hospital admissions, all retrieved from patients’ medical records, and the number of symptom-based exacerbations as assessed with TEXAS.
- Exacerbation-related self-management behavior, measured with TEXAS or the online questionnaire, and defined as taking 1 or more of the following 3 actions during symptom-based exacerbations: (1) contacting the health care professional, (2) starting a course of prednisolone and/or antibiotics, or (3) maximizing bronchodilator use. We also assessed the time between the date of exacerbation onset and the date of 1 of these 3 actions, defining actions taken within 3 days of exacerbation onset as adherence to the instructions.
- Exacerbation-related self-efficacy, measured with an exacerbation-related self-efficacy scale containing 5 questions. This questionnaire was created for the purpose of this study as, to our knowledge, no questionnaire existed that measured exacerbation-related self-efficacy. Reliability analyses showed a Cronbach alpha of .69 at baseline and .81 at follow-up.
- Health status, measured with (1) the Nijmegen Clinical Screening Instrument (NCSI), which is a battery of instruments measuring 8 subdomains of health status—subjective symptoms, dyspnea emotions, fatigue, behavioral impairment, subjective impairment, general quality of life (QoL), health-related QoL, and satisfaction with relationships [23]; (2) the Clinical Chronic Obstructive Pulmonary Disease Questionnaire (CCQ), which measures 3 subdomains, that is, symptoms, functional status, and mental status, resulting in a total score (24); and (3) the EuroQol-5 dimensions (EQ-5d), [24], which measures health-related quality of life, with a total score based on weighted scores on mobility, self-care, usual activities, pain/discomfort, and anxiety/depression as well as a vertical Visual Analogue Scale varying between 0 and 100.

At the start and at 12 months, data were gathered on exacerbation history, self-efficacy, and health status. CCQ and EQ-5d were also completed at 3, 6, and 9 months of follow-up.

At 12 months, information on health care utilization, lung function, respiratory medication use, and comorbid conditions was extracted from the participants’ medical records. In addition, all participants were asked to evaluate the supportive function of either the mHealth tool or the paper action plan by using a paper survey including closed-ended questions regarding the use, difficulty in use, and intended future use of the mHealth tool or the paper action plan. Besides, 3 questions were asked related to clarity, suitability, and follow-up of the advice given by the mHealth tool or the paper action plan. All questions included answers on a 7-point rating scale from strongly disagree (score 1) to strongly agree (score 7). The survey also included 1 question about frequency of usage at times of symptom worsening, with answers on a 7-point rating scale varying from 1=never to 7=always. In addition, participants of the intervention group were asked to complete the System Usability Scale (SUS) [25]. The SUS contains 10 questions on system usability, which are calculated into 1 total score between 0 and 100. SUS scores less than 68 are considered as low, greater than or equal to 68 and less than or equal to 80.3 as good, and greater than 80.3 as excellent.

### Sample Size Calculation and Statistical Analyses

Sample size calculation using analysis of variance showed that we needed 43 participants in each group for 80% power (alpha=.05, 2 sided) to detect an increase of 6 exacerbation-free weeks per year and anticipating a dropout rate of 20% (9/43). The calculation was based on a previous dataset [12] in which we found a mean of 44 exacerbation-free weeks, with SD 4.5 weeks. We used all available data from all participants based on the intention-to-treat principle. Missing data were not imputed.

We used the data recorded by the secured Web-based interface to assess the actual usage of the mHealth tool. We analyzed the answers to the paper evaluation survey to assess participants’ self-reported use of the mHealth tool and the paper action plan. Negative binomial regression analyses, controlling for follow-up time per participant, age and gender were used to analyze our primary outcome, that is, the number of exacerbation-free weeks, as well as the number of unscheduled health care contacts, self-reported exacerbations, exacerbations treated with antibiotics and/or prednisolone, and exacerbation-related hospital admissions.

To test the effect of the mHealth tool on the rate of symptom-based exacerbations and self-management behavior, we extracted exacerbation episodes from the TEXAS database. Each new episode was preceded by at least 2 exacerbation-free weeks or 2 weeks with missing data [22]. To assess patient delay in taking action when an exacerbation was imminent, the numbers of days were calculated between the date of exacerbation onset and the following actions: (1) the first date of contact with a health care professional, (2) starting date of the course of prednisolone and/or antibiotics, or (3) date of increase of bronchodilators. We categorized these variables into 2 groups: less than 3 days (according to instructions), and greater than or equal to 3 days. Separate multilevel logistic regression analyses were performed taking into account the clustering effect of exacerbations within patients and controlling for age and gender to examine (1) whether the mHealth tool led to higher percentages of self-management actions in case of an exacerbation compared with the paper action plan and (2) whether these actions were more often taken timely by patients in the intervention group compared with the control group.

We used generalized estimating equation regression analyses to estimate the effect of the mHealth tool on changes between baseline and follow-up scores of the self-efficacy scale, NCSI, CCQ, and EQ-5d compared with the paper action plan. We analyzed the CCQ and EQ-5d with all 5 measurement time points. We used 2-tailed *t* tests for independent samples and chi-square test to analyze the differences in the participants’ preferences between the mHealth tool and the paper action plan.

Statistical significance was assumed at *P*<.05 based on 2-sided tests. We used IBM SPSS Statistics 25 for the analyses.

## Results

### Patient Characteristics

**Figure 1:**
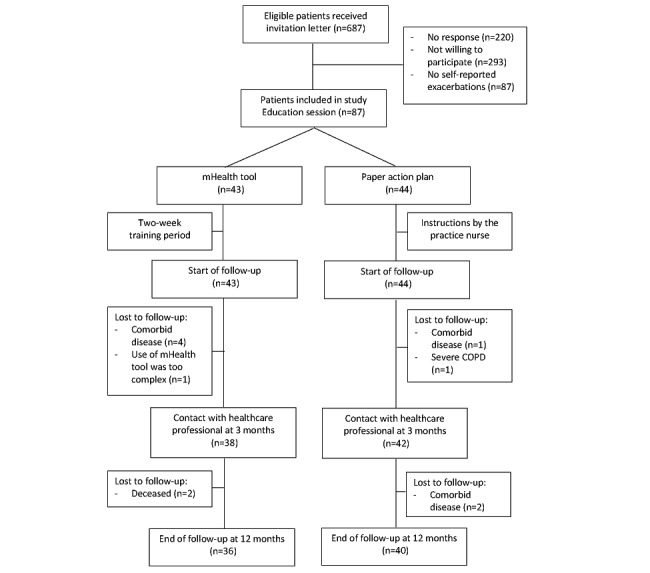
Flow diagram of the participants through the study. mHealth: mobile health.

Of the 87 patients included in the study, 43 were randomized to the intervention group. In addition, 45 patients were recruited from the hospitals and 42 from the general practices. Among them, 13% (11/87) dropped out of the study, 16% (7/43) in the intervention, and 9% (4/44) in the control group. A flowchart of the participants in the study is shown in Figure 1. Demographic and baseline characteristics are shown in Table 1.

Mean duration of follow-up was 48.1 (SD 11.7) weeks, and 11 COPD-related hospital admissions (6 in the intervention group and 5 in the control group) were reported as serious adverse events to the medical ethics review board.

### Usage of Mobile Health Tool and Paper Action Plan

From the Web-based interface, it appeared that 38 of the 43 patients (88%) in the mHealth group used the app 727 times in total during follow-up. No data on usage was available for 5 patients. The range in frequency of usage was 1 to 250 times with a median of 7 (25%-75% interquartile range was 3-14). Results of the evaluation questionnaire showed that more patients reported to have used their mHealth tool often (scores 6 and 7 on the 7-point rating scale) compared with patients in the control group who reported to have used their paper action plan (44.4% vs 17.2%, respectively).

**Table 1:** Baseline and demographic characteristics of the study population (N=87) per treatment arm.

Characteristic		Mobile health tool intervention group (n=43)	Paper action plan control group (n=44)
Recruited in hospital, n (%)		21 (49)	24 (55)
Follow-up in weeks, mean (SD)		48.3 (12.6)	49.8 (10.9)
Age (years), mean (SD)		69.3 (8.8)	65.9 (8.9)
Male sex, n (%)		25 (58)	29 (66)
Postbronchodilator FEV_1_^a^ (% predicted), mean (SD)		53.0 (21.5)	52.1 (19.8)
Medical Research Council dyspnea score, mean (SD)		2.5 (1.2)	2.6 (1.3)
Currently smoking, n (%)		13 (30)	11 (25)
Use of paper action plan prior to follow-up, n (%)		11 (26)	17 (39)
**Respiratory drug treatment, n (%)**			
	Long-acting bronchodilators	27 (63)	26 (59)
	Short-acting bronchodilators	30 (70)	31 (71)
	Inhaled corticosteroids (ICS)	7 (16)	13 (30)
	Long-acting bronchodilators + ICS	22 (51)	24 (55)
Low education level, n (%)		19 (44)	17 (39)
Diagnosis of COPD >5 years, n (%)		29 (68)	28 (64)
Currently working, n (%)		6 (14)	10 (23)
**Relevant comorbidities, n (%)**			
	Joint disorders	13 (30)	13 (30)
	Cardiac disorders	12 (28)	12 (27)
	Back pain	8 (19)	14 (32)
	Diabetes	3 (7)	3 (7)
	Depression and/or anxiety	3 (7)	2 (5)

^a^FEV_1_: forced expiratory volume in 1 second.

**Table 2:** Comparison of exacerbation-related outcomes between intervention group and control group (N=85).

Outcome	Mobile health tool intervention group (n=41), mean (SD)	Paper action plan control group (n=44), mean (SD)	Rate ratio (95% CI)^a^	*P* value
Exacerbation-free weeks^b^	30.6 (13.3)	28.0 (14.8)	1.21 (0.77-1.90)	.40
Unscheduled health care consultations because of respiratory complaints^c^	1.6 (1.7)	1.6 (2.0)	0.89 (0.50-1.60)	.70
Symptom-based exacerbations^b^	4.5 (2.3)	4.3 (2.1)	1.07 (0.65-1.75)	.80
Exacerbations treated with antibiotics and/or prednisolone^c^	1.1 (1.5)	1.0 (1.3)	1.01 (0.53-1.93)	.97
Exacerbation-related hospital admissions^c^	0.15 (0.43)	0.14 (0.41)	1.25 (0.35-4.44)	.74

^a^Calculated using negative binomial regression analyses, relative to participants’ follow-up time, controlling for age and gender.

^b^Data retrieved from weekly patient reports.

^c^Data retrieved from patient medical files.

### Exacerbation-Free Time and Other Exacerbation-Related Outcomes

Patients in the intervention group did not differ statistically significantly from patients in the control group in the number of weeks without exacerbations (mean 30.6 weeks, SD 13.3 weeks vs mean 28.0 weeks, SD 14.8 weeks, respectively; rate ratio [RR] 1.21; 95% CI 0.77-1.91; see Table 2). In addition, no statistically significant differences were found in the number of symptom-based exacerbations (mean 4.5, SD 2.3 vs mean 4.3, SD 2.1, respectively; RR 1.07, 95% CI 0.65-1.75), and the number of exacerbations treated with antibiotics and/or prednisolone (mean 1.0, SD 1.5 vs mean 1.0, SD 1.3, respectively; RR 1.01, 95% CI 0.53-1.93). Furthermore, no statistically significant between-group differences were found in the number of unscheduled health care contacts (mean 1.6, SD 1.7 vs mean 1.6, SD 2.0, respectively; RR 0.89, 95% CI 0.50-1.60) and exacerbation-related hospital admissions (mean 0.15, SD 0.43 vs mean 0.14, SD 0.41, respectively; RR 1.25, 95% CI 0.35-4.44).

### Self-Management Behavior

A total of 377 symptom-based exacerbation episodes were identified. Table 3 shows that there were no statistically significant differences between the intervention and control groups in the frequency of contacting a health care professional (odds ratio [OR] 0.94, 95% CI 0.51-1.73), initiating a course of antibiotics and/or prednisolone (OR 1.16, 95% CI 0.55-2.44), and increasing bronchodilator use (OR 1.08, 95% CI 0.56-2.06). We found no differences between the intervention and control groups in timely action, that is, contacting a health care professional (OR 2.21, 95% CI 0.78-6.23), starting a course of antibiotics and/or prednisolone (OR 1.46, 95% CI 0.48-4.42), or increasing bronchodilator use (OR 1.15, 95% CI 0.61-2.16) within 3 days of exacerbation onset (Table 4).

### Self-Efficacy and Health Status

We found no statistically significant difference in exacerbation-related self-efficacy between the intervention and control groups when comparing baseline scores with 12-month follow-up scores. In addition, there were no differences between the groups in changes between baseline and follow-up scores of the subscales of the NCSI, CCQ and EQ-5d (Table 5).

### Participant Evaluation of the Self-Management Support Tools

A total of 58 (67%) participants returned an evaluation form, of which 28 were in the intervention group. The mHealth tool was rated as a more useful support tool than the paper action plan (*P*=.02). No differences were found between the mHealth tool and the action plan in the self-reported frequency of use; in difficulty and future use of the tool; or in clarity, suitability, and follow-up of the advice. Overall, 26 participants of the intervention group completed all 10 questions of the SUS. The mean score was 78.5 (SD 14.4).

**Table 3:** Self-reported self-management behavior during exacerbation onset (N=377 exacerbations).

Self-management action	Mobile health tool intervention group (n=187), n (%)	Paper action plan control group (n=190), n (%)	Odds ratio (95% CI)^a^	*P* value
Contact health care professional	61 (32.6)	68 (35.8)	0.94 (0.51-1.73)	.83
Start prednisolone and/or antibiotics	64 (34.2)	62 (32.6)	1.16 (0.55-2.44)	.69
Increase bronchodilator use	135 (72.2)	135 (71.1)	1.08 (0.56-2.06)	.82

^a^Calculated using multilevel logistic regression analyses, including participant as cluster variable, controlling for age and gender.

**Table 4:** Self-reported self-management behavior within 3 days of exacerbation onset.

Self-management action	Mobile health tool intervention group		Paper action plan control group		Odds ratio (95% CI)^a^	*P* value
	n	<3 days, n (%)	n	<3 days, n (%)		
Contact health care professional	55	20 (36.4)	63	17 (27.0)	2.21 (0.78-6.23)	.13
Start prednisolone and/or antibiotics	58	23 (39.7)	57	23 (40.4)	1.46 (0.48-4.42)	.50
Increase bronchodilator use	122	87 (71.3)	117	80 (68.4)	1.15 (0.61-2.16)	.67

^a^Calculated using multilevel logistic regression analyses, including participant as cluster variable, controlling for age and gender.

**Table 5:** Baseline and follow-up scores of exacerbation-related self-efficacy and measures of health status.

Outcome		Mobile health tool intervention group		Paper action plan control group			*P* value
		Baseline (n=43), mean (SD)	12-month (n=35), mean (SD)	Baseline (n=44), mean (SD)	12-month (n=41), mean (SD)	Beta (95% CI)^a^	
Exacerbation-related self-efficacy^b^		2.91 (0.43)	2.98 (0.41)	2.84 (0.41)	2.87 (0.52)	0.03 (−0.17 to 0.22)	.91
**Health status measurements^c^**							
	NCSI^d^ QOL^e^	12.90 (8.32)	13.01 (8.27)	19.08 (11.93)	17.11 (12.14)	2.53 (−1.28 to −6.33)	.19
	NCSI HRQOL^f^	4.19 (1.74)	4.00 (1.61)	4.68 (1.78)	4.76 (1.93)	−0.16 (−0.89 to −0.57)	.66
	NCSI relationship	2.42 (0.93)	2.58 (0.87)	3.39 (1.74)	3.24 (1.59)	0.30 (−0.26 to −0.85)	.29
	NCSI subjective impairment	11.84 (5.80)	11.20 (4.12)	14.41 (6.57)	13.22 (6.57)	0.74 (−1.38 to −2.85)	.5
	NCSI behavioral impairment	22.11 (17.89)	20.50 (15.58)	19.11 (17.28)	20.43 (21.76)	−1.77 (−7.20 to −3.66)	.52
	NCSI subjective symptoms	9.86 (4.88)	9.23 (4.39)	11.91 (4.86)	11.05 (4.79)	0.35 (−1.40 to −2.11)	.69
	NCSI dyspnea emotions	8.77 (2.42)	8.71 (2.86)	11.59 (3.92)	10.73 (4.32)	0.85 (−0.58 to −2.27)	.24
	NCSI fatigue	35.93 (10.96)	35.23 (9.45)	37.32 (10.17)	37.73 (10.20)	−1.80 (−5.43 to −1.84)	.33
	CCQ^g^ total	2.06 (1.02)	1.84 (0.77)	2.31 (1.09)	2.16 (1.05)	−0.06 (−0.38 to −0.26)	.7
	CCQ symptoms	2.41 (1.12)	2.16 (0.80)	2.60 (1.27)	2.49 (1.24)	−0.22 (−0.67 to −0.23)	.34
	CCQ functional status	2.22 (1.38)	2.03 (1.21)	2.53 (1.36)	2.41 (1.32)	0.05 (−0.30 to −0.40)	.76
	CCQ mental status	1.03 (1.01)	0.81 (0.79)	1.30 (0.99)	1.00 (1.05)	0.09 (−0.34 to −0.53)	.68
	EQ-5d^h^	0.81 (0.15)	0.79 (0.16)	0.74 (0.20)	0.77 (0.21)	−0.05 (−0.13 to −0.03)	.22
	EQ VAS^i^	65.53 (17.37)	70.94 (12.92)	64.20 (15.35)	62.63 (19.14)	6.28 (−0.56 to −13.11)	.07

^a^Beta (B) indicates the difference between the intervention and control groups on differences between baseline and at 12 months.

^b^Higher score is positive.

^c^Higher score is negative.

^d^NCSI: Nijmegen Clinical Screening Instrument.

^e^QOL: quality of life.

^f^HRQOL: health-related quality of life.

^g^CCQ: Clinical Chronic Obstructive Pulmonary Disease Questionnaire.

^h^EQ-5d: EuroQol-5 dimensions.

^i^EQ VAS: EuroQol Visual Analogue Scale.

## Discussion

### Principal Findings

In this study, we examined the clinical effects of a smart mHealth tool to support COPD patients in the detection and treatment of exacerbations without the interference of a health care professional. Our primary hypothesis that the use of mHealth would lead to more weeks without exacerbations than care as usual, that is, the use of a paper action plan, was not confirmed. In addition, we did not find differences in exacerbation frequency, health care utilization, or self-management behavior between patients who used the mHealth tool and patients who used the paper action plan. Furthermore, patients using the tool did not report higher exacerbation-related self-efficacy or better health status than patients using a paper action plan. Patients evaluated the usability of the mHealth tool as good and considered it as more supportive than the action plan.

### Comparison With Previous Work

So far, studies on the effects of electronic health (eHealth) in the management of COPD have focused on the use of telemonitoring apps [26,27]. However, the mHealth tool in our study critically differs from telemonitoring tools as it enables the patient to be solely responsible for initiating treatment, without the interference of a health care professional. This impedes comparing our results with those found in telemonitoring trials. A recent Cochrane review showed that, until now, there is only limited evidence that interventions aimed at facilitating, supporting, and sustaining self‐management in patients with COPD and delivered via smart technology may improve outcomes such as health status and physical activity [28]. We found no effects of the mHealth tool on health status, measured with generic questionnaires, such as the NCSI and the EQ-5D, or with a disease-specific questionnaire, such as the CCQ.

In this study, we could not demonstrate positive effects on exacerbation-free weeks, exacerbation frequency, and health care utilization. Our primary outcome, *exacerbation-free weeks*, is directly related to exacerbation recovery time and may better reflect the burden of exacerbations in patients with COPD than exacerbation frequency does [21]. The mean number of exacerbation-free weeks we found during the 1-year follow-up period was 30.6 weeks in the intervention group and 28.0 weeks in the control group. These mean values are in line with the 33.4 weeks that we found in a previous study in which we examined the relationship between exacerbation frequency and exacerbation-free time in a cohort of 166 COPD patients [21]. These values suggest that there was enough room for improvement as the participants seem to suffer from symptom worsening in approximately 20 weeks in the 1-year follow-up period. Although patients rated the mHealth tool as a more useful support tool than the paper action plan, we found no differences between the mHealth tool and the paper action plan on self-management behavior and exacerbation-related self-efficacy. Previous studies showed that patients with COPD were able to use mHealth apps, including reporting daily symptoms and measuring physiological variables [29], and were able to interpret clinical data and use these within their self-management approach regardless of previous knowledge [30]. These findings are in line with our finding that participants in the intervention group rated the usability of the mHealth tool as good on average.

### Limitations

The major strength of this study is that we used a well-designed and validated mHealth tool to support self-management behavior [18]. In a qualitative study, Korpershoek et al demonstrated that to optimize engagement, mHealth interventions should be attractive, rewarding, safe, and tailored to the patient needs [31]. Our mHealth tool has been developed using feedback from patients with COPD, and its treatment advice can be tailored to the individual patient. To further optimize the use of the tool, patients were familiarized with the technique during a 2-week run-in period.

However, this study also has limitations. Two important limitations may have led to the statistically nonsignificant results of our primary outcome exacerbation-free weeks. First, we were surprised that the mean exacerbation-free time and its standard deviation at the 12-month follow-up differed substantially from the study data on which our power calculation was based [12]. We cannot explain this. As a result, the sample size in this study may have been too small to actually detect any statistically significant differences in our primary outcome. Conversely, when we performed our sample size calculation, there were no other studies available to provide data on exacerbation-free time. Second, offering both the intervention and control groups a short education session on the recognition and treatment of exacerbations before the start of the study and providing a paper action plan to the control group may have reduced the room for improvement and may have diluted potential differences between the 2 groups on our primary outcome exacerbation-free weeks. The purpose of the education session was to equalize the level of self-management knowledge among all participants and between both groups before the start of the trial. Our choice may have upgraded self-management knowledge and skills of all participants, although we did not measure what the participants had actually learned from the session to verify this assumption. We chose to provide our control group with a paper action plan according to the recommendations in the current national COPD guideline [19]; thereby supporting the self-management knowledge and skills of the control group participants. Many general practitioners and chest physicians in the Netherlands have not (yet) integrated the use of paper action plans in their daily practice. This was also found in our study, in which only 25.6% participants of the intervention group and 38.6% participants of the control group used a paper action plan before the study. Conversely, previous use of a paper action plan could have affected our primary outcome exacerbation-free weeks if patients in the intervention group continued to use the paper action plan instead of using the mHealth tool. Although we cannot refute this assumption with our data, we think that this effect (if any) has been small as we found that all intervention group participants used the mHealth tool and that more patients in the intervention group used the mHealth tool than patients in the control group used their paper plans.

There are also other limitations. Of the 467 patients that responded to the study invitation and met the inclusion criteria, 283 were not willing to participate. This may have led to selection bias. We believe that the risk of contamination because of the individual randomization procedure is negligible, as both the mHealth tool and the paper action plan were used at home, outside the reach of the health care professional. Although in the last 4 months of the trial we had to switch to collecting exacerbation-related outcomes through a digital survey tool instead of the automated TEXAS telephone calls [22], this did not impact the rate of data entry, and the participants experienced no difficulties. So, although it was an undesirable deviation from the protocol, we have no concerns on the reliability of the primary outcome data. Finally, 12 months of follow-up may have been too short for the mHealth tool to reach its maximum effect, as it may take more time to sustain engagement with the technology and change self-management behavior [28].

### Future Research

Although we were not able to demonstrate positive effects of our mHealth tool, we still believe that the use of eHealth tools, including machine learning techniques, better suits the goals of patient-centered care and self-management support than telemonitoring tools where a health care professional monitors the patient from a distance. Besides, in this study, the patients using the mHealth tool evaluated it as usable and more supportive than patients using a conventional supportive tool, that is, the written paper action plan. Future research should focus more on patients who are specifically interested in using digital tools in their daily life, as these patients may have greater benefit from them [28]. More information on patient perceptions of the use of the mHealth tool is also needed and could be collected by patient interviews or open-ended surveys. In addition, more research is needed on the clinical effects of the mHealth tool when used appropriately and the factors that are associated with appropriate use. However, for these purposes, there is a need for studies with larger patient populations than in this study.

### Conclusions

In this study, we examined the effectiveness of an mHealth tool designed to support COPD patients in their self-management of symptom worsening to reduce the impact of exacerbations. The app was not designed to replace the health care professional but to reduce patient delay. Patients evaluated the app’s usability as good and as more supportive than the paper action plan. Although this study did not show beneficial effects of the mHealth app compared with the use of a paper action plan, based on patient’s preference, it may be a valuable alternative to a paper action plan in the management of COPD.

## Appendix

Multimedia Appendix 1 Contents of the mobile health tool.

Multimedia Appendix 2 CONSORT-EHEALTH checklist (V 1.6.1).

## Abbreviations

CCQ: Clinical Chronic Obstructive Pulmonary Disease Questionnaire
COPD: chronic obstructive pulmonary disease
eHealth: electronic health
EQ-5D: EuroQol-5 dimensions
FEV1: forced expiratory volume in 1 second
mHealth: mobile health
NCSI: Nijmegen Clinical Screening Instrument
OR: odds ratio
QoL: quality of life
RR: rate ratio
SUS: System Usability Scale
TEXAS: Telephonic Exacerbation Assessment System
