# Supplementary material for: A Smart Mobile Health Tool Versus a Paper Action Plan to Support Self-Management of Chronic Obstructive Pulmonary Disease Exacerbations: Randomized Controlled Trial
Source: JMIR Mhealth Uhealth. 2019 Oct 9;7(10):e14408. doi: 10.2196/14408 (PMC6811767; doi:10.2196/14408)
Supplement: Multimedia Appendix 1 [file mhealth_v7i10e14408_app1.pdf]

Multimedia Appendix 1. Contents of the mHealth tool.

**Questions on symptoms and medication use**<br><br>**In comparison to normal, do you today …………………….**	**Answers**
1. ..experience more shortness of breath?	YES/NO
2. ..experience more fear because of your shortness of breath?	YES/NO
3. ..experience more fatigue?	YES/NO
4. ..feel more hindered by your COPD during your daily activities?	YES/NO
5. ..experience more sputum in you airways?	YES/NO
6. ..notice any difference in your sputum colour/composition?	YES/NO
7. ..experience more wheezing?	YES/NO
8. ..experience more coughing?	YES/NO
9. ..have a sore throat?	YES/NO
10. ..have a cold or a runny nose?	YES/NO
11. ..experience more stress or tension?	YES/NO
12. ..have used more of your bronchodilators?	YES/NO
**Questions on physiological measurements**	
1. What is your temperature in degrees Celsius?	_ _._ (Example: 37.2)
2. What is your FEV1 in litres?	_._ _ (Example: 1.31)
3. What is your SpO2 in percentages?	_ _ (Example: 95)
