# Supplementary material for: A Smart Mobile Health Tool Versus a Paper Action Plan to Support Self-Management of Chronic Obstructive Pulmonary Disease Exacerbations: Randomized Controlled Trial
Source: JMIR Mhealth Uhealth. 2019 Oct 9;7(10):e14408. doi: 10.2196/14408 (PMC6811767; doi:10.2196/14408)
Supplement: Multimedia Appendix 2 [file mhealth_v7i10e14408_app2.pdf]

CONSORT-EHEALTH (V 1.6.1) - Submission/Publication Form

The CONSORT-EHEALTH checklist is intended for authors of randomized trials evaluating web-based and Internet-based applications/interventions, including mobile interventions, electronic games (incl multiplayer games), social media, certain telehealth applications, and other interactive and/or networked electronic applications. Some of the items (e.g. all subitems under item 5 - description of the intervention) may also be applicable for other study designs.

The goal of the CONSORT EHEALTH checklist and guideline is to be
a) a guide for reporting for authors of RCTs,
b) to form a basis for appraisal of an ehealth trial (in terms of validity)

CONSORT-EHEALTH items/subitems are MANDATORY reporting items for studies published in the Journal of Medical Internet Research and other journals / scientific societies endorsing the checklist.

Items numbered 1., 2., 3., 4a., 4b etc are original CONSORT or CONSORT-NPT (non-pharmacologic treatment) items.
Items with Roman numerals (i., ii, iii, iv etc.) are CONSORT-EHEALTH extensions/clarifications.

As the CONSORT-EHEALTH checklist is still considered in a formative stage, we would ask that you also RATE ON A SCALE OF 1-5 how important/useful you feel each item is FOR THE PURPOSE OF THE CHECKLIST and reporting guideline (optional).

Mandatory reporting items are marked with a red *.
In the textboxes, either copy & paste the relevant sections from your manuscript into this form - please include any quotes from your manuscript in QUOTATION MARKS,
or answer directly by providing additional information not in the manuscript, or elaborating on why the item was not relevant for this study.

YOUR ANSWERS WILL BE PUBLISHED AS A SUPPLEMENTARY FILE TO YOUR PUBLICATION IN JMIR AND ARE CONSIDERED PART OF YOUR PUBLICATION (IF ACCEPTED).
Please fill in these questions diligently. Information will not be copyedited, so please use proper spelling and grammar, use correct capitalization, and avoid abbreviations.

DO NOT FORGET TO SAVE AS PDF _AND_ CLICK THE SUBMIT BUTTON SO YOUR ANSWERS ARE IN OUR DATABASE !!!

Citation Suggestion (if you append the pdf as Appendix we suggest to cite this paper in the caption):
Eysenbach G, CONSORT-EHEALTH Group
CONSORT-EHEALTH: Improving and Standardizing Evaluation Reports of Web-based and Mobile Health Interventions
J Med Internet Res 2011;13(4):e126
URL: http://www.jmir.org/2011/4/e126/
doi: 10.2196/jmir.1923
PMID: 22209829

*Vereist

Your name *

First Last

Erik Bischoff

Primary Affiliation (short), City, Country *

University of Toronto, Toronto, Canada

Radboud university medical center

Your e-mail address *

abc@gmail.com

erik.bischoff@radboudumc.nl

Title of your manuscript *
Provide the (draft) title of your manuscript.

A smart mHealth tool versus a paper action plan to support self-management of COPD exacerbations: a randomized controlled trial

Name of your App/Software/Intervention *
If there is a short and a long/alternate name, write the short name first and add the long name in brackets.

mHealth tool to support COPD exacerba

Evaluated Version (if any)
e.g. "V1", "Release 2017-03-01", "Version 2.0.27913"

Jouw antwoord

Language(s) *
What language is the intervention/app in? If multiple languages are available, separate by comma (e.g. "English, French")

Dutch, English

URL of your Intervention Website or App
e.g. a direct link to the mobile app on app in appstore (itunes, Google Play), or URL of the website. If the intervention is a DVD or hardware, you can also link to an Amazon page.

https://www.monitair.com

URL of an image/screenshot (optional)

Jouw antwoord

Accessibility *
Can an enduser access the intervention presently?

- ○ access is free and open
- ● access only for special usergroups, not open
- ○ access is open to everyone, but requires payment/subscription/in-app purchases
- ○ app/intervention no longer accessible
- ○ Anders:

Primary Medical Indication/Disease/Condition *
e.g. "Stress", "Diabetes", or define the target group in brackets after the condition, e.g. "Autism (Parents of children with)", "Alzheimers (Informal Caregivers of)"

COPD exacerbations

Primary Outcomes measured in trial *
comma-separated list of primary outcomes reported in the trial

exacerbation-free weeks;

Secondary/other outcomes
Are there any other outcomes the intervention is expected to affect?

health status, self-efficacy, self-management behaviour, healthcare utilization, and usability

Recommended "Dose" *
What do the instructions for users say on how often the app should be used?

- ○ Approximately Daily
- ○ Approximately Weekly
- ○ Approximately Monthly
- ○ Approximately Yearly
- ◉ "as needed"
- ○ Anders:

Approx. Percentage of Users (starters) still using the app as recommended after 3 months *

- ◉ unknown / not evaluated
- ○ 0-10%
- ○ 11-20%
- ○ 21-30%
- ○ 31-40%
- ○ 41-50%
- ○ 51-60%
- ○ 61-70%
- ○ 71%-80%
- ○ 81-90%
- ○ 91-100%
- ○ Anders:

Overall, was the app/intervention effective? *

- ○ yes: all primary outcomes were significantly better in intervention group vs control
- ○ partly: SOME primary outcomes were significantly better in intervention group vs control
- ◉ no statistically significant difference between control and intervention
- ○ potentially harmful: control was significantly better than intervention in one or more outcomes
- ○ inconclusive: more research is needed
- ○ Anders:

Article Preparation Status/Stage *

At which stage in your article preparation are you currently (at the time you fill in this form)

- ○ not submitted yet - in early draft status
- ○ not submitted yet - in late draft status, just before submission
- ◉ submitted to a journal but not reviewed yet
- ○ submitted to a journal and after receiving initial reviewer comments
- ○ submitted to a journal and accepted, but not published yet
- ○ published
- ○ Anders:

Journal *

If you already know where you will submit this paper (or if it is already submitted), please provide the journal name (if it is not JMIR, provide the journal name under "other")

- ○ not submitted yet / unclear where I will submit this
- ◉ Journal of Medical Internet Research (JMIR)
- ○ JMIR mHealth and UHealth
- ○ JMIR Serious Games
- ○ JMIR Mental Health
- ○ JMIR Public Health
- ○ JMIR Formative Research
- ○ Other JMIR sister journal
- ○ Anders:

Is this a full powered effectiveness trial or a pilot/feasibility trial? *

- ◉ Pilot/feasibility
- ○ Fully powered

Manuscript tracking number *

If this is a JMIR submission, please provide the manuscript tracking number under "other" (The ms tracking number can be found in the submission acknowledgement email, or when you login as author in JMIR. If the paper is already published in JMIR, then the ms tracking number is the four-digit number at the end of the DOI, to be found at the bottom of each published article in JMIR)

- ◉ no ms number (yet) / not (yet) submitted to / published in JMIR
- ○ Anders:

TITLE AND ABSTRACT

1a) TITLE: Identification as a randomized trial in the title

1a) Does your paper address CONSORT item 1a? *

I.e does the title contain the phrase "Randomized Controlled Trial"? (if not, explain the reason under "other")

- (x) yes
- ( ) Anders:

1a-i) Identify the mode of delivery in the title

Identify the mode of delivery. Preferably use "web-based" and/or "mobile" and/or "electronic game" in the title. Avoid ambiguous terms like "online", "virtual", "interactive". Use "Internet-based" only if Intervention includes non-web-based Internet components (e.g. email), use "computer-based" or "electronic" only if offline products are used. Use "virtual" only in the context of "virtual reality" (3-D worlds). Use "online" only in the context of "online support groups". Complement or substitute product names with broader terms for the class of products (such as "mobile" or "smart phone" instead of "iphone"), especially if the application runs on different platforms.

subitem not at all important ( ) ( ) ( ) ( ) (x) essential

Does your paper address subitem 1a-i? *

Copy and paste relevant sections from manuscript title (include quotes in quotation marks "like this" to indicate direct quotes from your manuscript), or elaborate on this item by providing additional information not in the ms, or briefly explain why the item is not applicable/relevant for your study

"smart mHealth tool"

1a-ii) Non-web-based components or important co-interventions in title

Mention non-web-based components or important co-interventions in title, if any (e.g., "with telephone support").

subitem not at all important (x) ( ) ( ) ( ) ( ) essential

Does your paper address subitem 1a-ii?

Copy and paste relevant sections from manuscript title (include quotes in quotation marks "like this" to indicate direct quotes from your manuscript), or elaborate on this item by providing additional information not in the ms, or briefly explain why the item is not applicable/relevant for your study

subitem not relevant, subitem 1a-i covers the mode of delivery

1a-iii) Primary condition or target group in the title

Mention primary condition or target group in the title, if any (e.g., "for children with Type I Diabetes") Example: A Web-based and Mobile Intervention with Telephone Support for Children with Type I Diabetes: Randomized Controlled Trial

subitem not at all important ( ) ( ) ( ) ( ) (x) essential

Does your paper address subitem 1a-iii? *

Copy and paste relevant sections from manuscript title (include quotes in quotation marks "like this" to indicate direct quotes from your manuscript), or elaborate on this item by providing additional information not in the ms, or briefly explain why the item is not applicable/relevant for your study

"to support self-management of COPD exacerbations"

1b) ABSTRACT: Structured summary of trial design, methods, results, and conclusions

NPT extension: Description of experimental treatment, comparator, care providers, centers, and blinding status.

1b-i) Key features/functionalities/components of the intervention and comparator in the METHODS section of the ABSTRACT

Mention key features/functionalities/components of the intervention and comparator in the abstract. If possible, also mention theories and principles used for designing the site. Keep in mind the needs of systematic reviewers and indexers by including important synonyms. (Note: Only report in the abstract what the main paper is reporting. If this information is missing from the main body of text, consider adding it)

subitem not at all important ○ ○ ○ ○ ◉ essential

Does your paper address subitem 1b-i? *

Copy and paste relevant sections from the manuscript abstract (include quotes in quotation marks "like this" to indicate direct quotes from your manuscript), or elaborate on this item by providing additional information not in the ms, or briefly explain why the item is not applicable/relevant for your study

"To examine the effects of a smart mHealth tool that supports COPD patients in the self-management of exacerbations by providing predictions of early exacerbation onset and timely treatment advices without the interference of healthcare professionals."
Methods
"In a multicenter, two-arm randomized controlled trial (RCT) with 12-months follow-up patients with COPD used the smart mHealth tool (intervention group) or a paper action plan (control group) when they experienced worsening of respiratory symptoms."

1b-ii) Level of human involvement in the METHODS section of the ABSTRACT

Clarify the level of human involvement in the abstract, e.g., use phrases like "fully automated" vs. "therapist/nurse/care provider/physician-assisted" (mention number and expertise of providers involved, if any). (Note: Only report in the abstract what the main paper is reporting. If this information is missing from the main body of text, consider adding it)

subitem not at all important ○ ○ ○ ○ ◉ essential

Does your paper address subitem 1b-ii?

Copy and paste relevant sections from the manuscript abstract (include quotes in quotation marks "like this" to indicate direct quotes from your manuscript), or elaborate on this item by providing additional information not in the ms, or briefly explain why the item is not applicable/relevant for your study

"without the interference of healthcare professionals."

1b-iii) Open vs. closed, web-based (self-assessment) vs. face-to-face assessments in the METHODS section of the ABSTRACT

Mention how participants were recruited (online vs. offline), e.g., from an open access website or from a clinic or a closed online user group (closed usergroup trial), and clarify if this was a purely web-based trial, or there were face-to-face components (as part of the intervention or for assessment). Clearly say if outcomes were self-assessed through questionnaires (as common in web-based trials). Note: In traditional offline trials, an open trial (open-label trial) is a type of clinical trial in which both the researchers and participants know which treatment is being administered. To avoid confusion, use "blinded" or "unblinded" to indicated the level of blinding instead of "open", as "open" in web-based trials usually refers to "open access" (i.e. participants can self-enrol). (Note: Only report in the abstract what the main paper is reporting. If this information is missing from the main body of text, consider adding it)

subitem not at all important ○ ○ ○ ◉ ○ essential

Does your paper address subitem 1b-iii?

Copy and paste relevant sections from the manuscript abstract (include quotes in quotation marks "like this" to indicate direct quotes from your manuscript), or elaborate on this item by providing additional information not in the ms, or briefly explain why the item is not applicable/relevant for your study

"Of the 87 patients with COPD recruited from primary and secondary care, 43 were randomized to the intervention group. "

1b-iv) RESULTS section in abstract must contain use data

Report number of participants enrolled/assessed in each group, the use/uptake of the intervention (e.g., attrition/adherence metrics, use over time, number of logins etc.), in addition to primary/secondary outcomes. (Note: Only report in the abstract what the main paper is reporting. If this information is missing from the main body of text, consider adding it)

subitem not at all important ○ ○ ○ ◉ ○ essential

Does your paper address subitem 1b-iv?

Copy and paste relevant sections from the manuscript abstract (include quotes in quotation marks "like this" to indicate direct quotes from your manuscript), or elaborate on this item by providing additional information not in the ms, or briefly explain why the item is not applicable/relevant for your study

"Patients using the mHealth tool valued it as a more supportive tool than patients using the paper action plan. Patients considered the usability of the mHealth tool as good."

1b-v) CONCLUSIONS/DISCUSSION in abstract for negative trials

Conclusions/Discussions in abstract for negative trials: Discuss the primary outcome - if the trial is negative (primary outcome not changed), and the intervention was not used, discuss whether negative results are attributable to lack of uptake and discuss reasons. (Note: Only report in the abstract what the main paper is reporting. If this information is missing from the main body of text, consider adding it)

subitem not at all important ○ ○ ○ ◉ ○ essential

Does your paper address subitem 1b-v?

Copy and paste relevant sections from the manuscript abstract (include quotes in quotation marks "like this" to indicate direct quotes from your manuscript), or elaborate on this item by providing additional information not in the ms, or briefly explain why the item is not applicable/relevant for your study

This is a negative trial. The abstract only concludes about the results, the discussion section in the main paper dwells about the primary outcome and the uptake.

INTRODUCTION

2a) In INTRODUCTION: Scientific background and explanation of rationale

2a-i) Problem and the type of system/solution

Describe the problem and the type of system/solution that is object of the study: intended as stand-alone intervention vs. incorporated in broader health care program? Intended for a particular patient population? Goals of the intervention, e.g., being more cost-effective to other interventions, replace or complement other solutions? (Note: Details about the intervention are provided in "Methods" under 5)

subitem not at all important ○ ○ ○ ○ ◉ essential

Does your paper address subitem 2a-i? *

Copy and paste relevant sections from the manuscript (include quotes in quotation marks "like this" to indicate direct quotes from your manuscript), or elaborate on this item by providing additional information not in the ms, or briefly explain why the item is not applicable/relevant for your study

"Telemonitoring, in which patients record and send information on symptoms and/or physiological measurements to a supervising clinician, may be an alternative approach to self-management strategies to reduce the impact of COPD exacerbations. Beneficial effects have been reported on number of hospital admissions,[13] emergency visits,[13] and quality of life in some studies,[14, 15] but not in all.[16] There is much heterogeneity between telemonitoring interventions regarding devices and clinical content, and the amount of additional support that patients receive. However, in contrast with self-management, telemonitoring strongly depends on the judgement of the clinician and the patient is not expected to interpret his or her own symptoms and signs. Therefore, we have developed [17] and validated [18] an innovative mHealth tool, called the Adaptive Computerised COPD Exacerbation Self-management Support (ACCESS) system. This tool aims to tailor self-management support more efficiently and continuously than with a written action plan, but without heavily increasing the involvement of healthcare professionals to monitor input as is the case with telemonitoring. The mHealth tool integrates information on symptom changes and physiological measurements (i.e., pulse oximetry, spirometry and measurement of body temperature) in an easy-to-use application by means of a smartphone.[17] Based on a decision tree built by a clinical expert panel and a Bayesian prediction model, the tool provides automated, tailored self-management advices to the patient without the involvement of the healthcare professional.[18] Patients can use the tool at their own initiative to monitor symptom changes at any time of day or night, and receive ad hoc, tailored advice. "

2a-ii) Scientific background, rationale: What is known about the (type of) system

Scientific background, rationale: What is known about the (type of) system that is the object of the study (be sure to discuss the use of similar systems for other conditions/diagnoses, if appropiate), motivation for the study, i.e. what are the reasons for and what is the context for this specific study, from which stakeholder viewpoint is the study performed, potential impact of findings [2]. Briefly justify the choice of the comparator.

subitem not at all important ○ ○ ◉ ○ ○ essential

Does your paper address subitem 2a-ii? *

Copy and paste relevant sections from the manuscript (include quotes in quotation marks "like this" to indicate direct quotes from your manuscript), or elaborate on this item by providing additional information not in the ms, or briefly explain why the item is not applicable/relevant for your study

"Telemonitoring, in which patients record and send information on symptoms and/or physiological measurements to a supervising clinician, may be an alternative approach to self-management strategies to reduce the impact of COPD exacerbations. Beneficial effects have been reported on number of hospital admissions,[13] emergency visits,[13] and quality of life in some studies,[14, 15] but not in all.[16] There is much heterogeneity between telemonitoring interventions regarding devices and clinical content, and the amount of additional support that patients receive. However, in contrast with self-management, telemonitoring strongly depends on the judgement of the clinician and the patient is not expected to interpret his or her own symptoms and signs. Therefore, we have developed [17] and validated [18] an innovative mHealth tool, called the Adaptive Computerised COPD Exacerbation Self-management Support (ACCESS) system. This tool aims to tailor self-management support more efficiently and continuously than with a written action plan, but without heavily increasing the involvement of healthcare professionals to monitor input as is the case with telemonitoring. The mHealth tool integrates information on symptom changes and physiological measurements (i.e., pulse oximetry, spirometry and measurement of body temperature) in an easy-to-use application by means of a smartphone.[17] Based on a decision tree built by a clinical expert panel and a Bayesian prediction model, the tool provides automated, tailored self-management advices to the patient without the involvement of the healthcare professional.[18] Patients can use the tool at their own initiative to monitor symptom changes at any time of day or night, and receive ad hoc, tailored advice. "

2b) In INTRODUCTION: Specific objectives or hypotheses

Does your paper address CONSORT subitem 2b? *

Copy and paste relevant sections from the manuscript (include quotes in quotation marks "like this" to indicate direct quotes from your manuscript), or elaborate on this item by providing additional information not in the ms, or briefly explain why the item is not applicable/relevant for your study

"In this study, we examined the clinical effectiveness of the mHealth tool. We hypothesised that in patients with COPD the use of the tool would lead to more weeks without exacerbations, improvement in health status, self-efficacy, and self-management behaviour, and a reduction in healthcare utilization, compared to the use of a paper exacerbation action plan. We also evaluated how patients valued the tool's supportive function and usability. "

METHODS

3a) Description of trial design (such as parallel, factorial) including allocation ratio

Does your paper address CONSORT subitem 3a? *

Copy and paste relevant sections from the manuscript (include quotes in quotation marks "like this" to indicate direct quotes from your manuscript), or elaborate on this item by providing additional information not in the ms, or briefly explain why the item is not applicable/relevant for your study

"This study was a multicenter, parallel, two-arm, randomized controlled trial (RCT) with a follow-up of 12 months per patient. "

3b) Important changes to methods after trial commencement (such as eligibility criteria), with reasons

Does your paper address CONSORT subitem 3b? *

Copy and paste relevant sections from the manuscript (include quotes in quotation marks "like this" to indicate direct quotes from your manuscript), or elaborate on this item by providing additional information not in the ms, or briefly explain why the item is not applicable/relevant for your study

not applicable

3b-i) Bug fixes, Downtimes, Content Changes

Bug fixes, Downtimes, Content Changes: ehealth systems are often dynamic systems. A description of changes to methods therefore also includes important changes made on the intervention or comparator during the trial (e.g., major bug fixes or changes in the functionality or content) (5-iii) and other "unexpected events" that may have influenced study design such as staff changes, system failures/downtimes, etc. [2].

subitem not at all important ◉ ○ ○ ○ ○ essential

Does your paper address subitem 3b-i?

Copy and paste relevant sections from the manuscript (include quotes in quotation marks "like this" to indicate direct quotes from your manuscript), or elaborate on this item by providing additional information not in the ms, or briefly explain why the item is not applicable/relevant for your study

not applicable

4a) Eligibility criteria for participants

Does your paper address CONSORT subitem 4a? *

Copy and paste relevant sections from the manuscript (include quotes in quotation marks "like this" to indicate direct quotes from your manuscript), or elaborate on this item by providing additional information not in the ms, or briefly explain why the item is not applicable/relevant for your study

"Patients were eligible for participation if they (i) were at least 40 years of age, (ii) had a spirometry-confirmed diagnosis of COPD (post-bronchodilator forced expiratory volume in one second / forced vital capacity (FEV1/FVC) < 0.7), and (iii) had experienced two or more symptom-based exacerbations in the previous 12 months, defined as a change for ≥ 2 consecutive days in either ≥ 2 major symptoms (dyspnoea, sputum purulence, sputum amount) or any 1 major symptom plus ≥ 1 minor symptoms (colds, wheeze, sore throat, cough).[3, 20] Exclusion criteria were (i) severe co-morbid conditions that prohibited safe participation, (ii) insufficient knowledge of the Dutch language, and (iii) persisting difficulties in using the mHealth system after a two-week practice period and additional assistance. "

4a-i) Computer / Internet literacy

Computer / Internet literacy is often an implicit "de facto" eligibility criterion - this should be explicitly clarified.

subitem not at all important ○ ○ ○ ◉ ○ essential

Does your paper address subitem 4a-i?

Copy and paste relevant sections from the manuscript (include quotes in quotation marks "like this" to indicate direct quotes from your manuscript), or elaborate on this item by providing additional information not in the ms, or briefly explain why the item is not applicable/relevant for your study

"persisting difficulties in using the mHealth system after a two-week practice period and additional assistance. "

4a-ii) Open vs. closed, web-based vs. face-to-face assessments:

Open vs. closed, web-based vs. face-to-face assessments: Mention how participants were recruited (online vs. offline), e.g., from an open access website or from a clinic, and clarify if this was a purely web-based trial, or there were face-to-face components (as part of the intervention or for assessment), i.e., to what degree got the study team to know the participant. In online-only trials, clarify if participants were quasi-anonymous and whether having multiple identities was possible or whether technical or logistical measures (e.g., cookies, email confirmation, phone calls) were used to detect/prevent these.

subitem not at all important ○ ○ ○ ◉ ○ essential

Does your paper address subitem 4a-ii? *

Copy and paste relevant sections from the manuscript (include quotes in quotation marks "like this" to indicate direct quotes from your manuscript), or elaborate on this item by providing additional information not in the ms, or briefly explain why the item is not applicable/relevant for your study

"Due to the type of intervention, patients and healthcare professionals could not be blinded for group assignment. Also, the research team could not be blinded, since it was responsible for the personalisation and technical support of the mHealth tool. The study statistician (RA), who was responsible for analysing the data, was blinded for study assignment of the participants until the analyses had been finished. "

4a-iii) Information giving during recruitment

Information given during recruitment. Specify how participants were briefed for recruitment and in the informed consent procedures (e.g., publish the informed consent documentation as appendix, see also item X26), as this information may have an effect on user self-selection, user expectation and may also bias results.

subitem not at all important ○ ○ ○ ◉ ○ essential

Does your paper address subitem 4a-iii?

Copy and paste relevant sections from the manuscript (include quotes in quotation marks "like this" to indicate direct quotes from your manuscript), or elaborate on this item by providing additional information not in the ms, or briefly explain why the item is not applicable/relevant for your study

Information was given by patients' Healthcare professional. Informed consent was signed after group meetings in which purpose of study and participant contribution was explained

4b) Settings and locations where the data were collected

Does your paper address CONSORT subitem 4b? *

Copy and paste relevant sections from the manuscript (include quotes in quotation marks "like this" to indicate direct quotes from your manuscript), or elaborate on this item by providing additional information not in the ms, or briefly explain why the item is not applicable/relevant for your study

"Our primary outcome was the difference in number of exacerbation-free weeks between the intervention and control groups. An exacerbation-free week was defined as a week in which there had not been episodes of two or more consecutive days with worsening of two major symptoms (i.e. dyspnoea, sputum purulence, sputum amount) or one major and one or more minor symptoms (i.e. colds, wheeze, sore throat, and cough).[21] Symptom changes were assessed by means of the Telephonic Exacerbation Assessment System (TEXAS), an automated telephone call system that contacted participants weekly on the day and time of their preference.[22] TEXAS consisted of closed questions regarding changes in respiratory symptoms, use of healthcare resources and use of respiratory medication in the week prior to the call and its validity has been demonstrated previously.[22] Due to discontinuation of the contract with the provider of TEXAS, the last 19 participants in the trial received a weekly online questionnaire containing the same questions as with TEXAS. These participants used both measuring tools during two weeks before stopping with TEXAS, which enabled us to compare data entry from TEXAS with the online survey tool. We found no differences in data entry.
Secondary outcomes included:

- Exacerbation-related outcomes, i.e. the number of unscheduled healthcare contacts, the number of exacerbations treated with antibiotics and/or prednisolone, and the number of exacerbation-related hospital admissions, all retrieved from patients' medical records, and the number of symptom-based exacerbations as assessed with TEXAS.
- Exacerbation-related self-management behaviour, measured with TEXAS or the online questionnaire, and defined as taken one or more of the following three actions during symptom-based exacerbations: (i) contacting the healthcare professional , (ii) starting a course of prednisolone and/or antibiotics, or (iii) maximising bronchodilator use. We also assessed the time between the date of exacerbation onset and the date of one of these three actions, defining actions taken within 3 days of exacerbation onset as adherence to the instructions.
- Exacerbation-related self-efficacy, measured with an exacerbation-related self-efficacy scale containing 5 questions. This questionnaire was created for the purpose of this study, since to our knowledge no questionnaire existed that measured exacerbation-related self-efficacy. Reliability analyses showed a Cronbach's alpha of 0.69 at baseline and 0.81 at follow-up.
- Health status, measured with (i) the Nijmegen Clinical Screening Instrument (NCSI), which is a battery of instruments measuring eight sub-domains of health status –subjective symptoms, dyspnoea emotions, fatigue, behavioural impairment, subjective impairment, general quality of life (QoL), health-related QoL and satisfaction with relationships [23], (ii) the Clinical COPD Questionnaire (CCQ), which measures 3 sub-domains, i.e. symptoms, functional status and mental status, resulting in a total score (24); and (iii) the EuroQol-5 dimensions (EQ-5d),[24] which measures health-related quality of life with a total score based on weighted scores on mobility, self-care, usual activities, pain/discomfort and anxiety/depression, as well as a vertical VAS scale varying between 0 and 100.

At the start and at 12 months, data were gathered on exacerbation history, self-efficacy and health status. CCQ and EQ-5d were also completed at three, six, and nine months follow-up.

At 12 months, information on healthcare utilization, lung function, respiratory medication use, and comorbid conditions were extracted from the participants' medical records. Also, all participants were asked to evaluate the supportive function of either the mHealth tool or the paper action plan and participants of the intervention group were asked to complete the System Usability Scale (SUS). [25] The SUS contains 10 questions on system usability, which are calculated into one total score between 0 and 100. SUS scores < 68 are considered as low, ≥ 68 and ≤ 80.3 as good, and > 80.3 as excellent.
"

4b-i) Report if outcomes were (self-)assessed through online questionnaires

Clearly report if outcomes were (self-)assessed through online questionnaires (as common in web-based trials) or otherwise.

subitem not at all important ○ ○ ◉ ○ ○ essential

Does your paper address subitem 4b-i? *

Copy and paste relevant sections from the manuscript (include quotes in quotation marks "like this" to indicate direct quotes from your manuscript), or elaborate on this item by providing additional information not in the ms, or briefly explain why the item is not applicable/relevant for your study

"Due to discontinuation of the contract with the provider of TEXAS, the last 19 participants in the trial received a weekly online questionnaire containing the same questions as with TEXAS. These participants used both measuring tools during two weeks before stopping with TEXAS, which enabled us to compare data entry from TEXAS with the online survey tool. We found no differences in data entry. "

4b-ii) Report how institutional affiliations are displayed

Report how institutional affiliations are displayed to potential participants [on ehealth media], as affiliations with prestigious hospitals or universities may affect volunteer rates, use, and reactions with regards to an intervention.(Not a required item – describe only if this may bias results)

subitem not at all important ◉ ○ ○ ○ ○ essential

Does your paper address subitem 4b-ii?

Copy and paste relevant sections from the manuscript (include quotes in quotation marks "like this" to indicate direct quotes from your manuscript), or elaborate on this item by providing additional information not in the ms, or briefly explain why the item is not applicable/relevant for your study

not applicable

5) The interventions for each group with sufficient details to allow replication, including how and when they were actually administered

5-i) Mention names, credential, affiliations of the developers, sponsors, and owners

Mention names, credential, affiliations of the developers, sponsors, and owners [6] (if authors/evaluators are owners or developer of the software, this needs to be declared in a "Conflict of interest" section or mentioned elsewhere in the manuscript).

subitem not at all important ○ ◉ ○ ○ ○ essential

Does your paper address subitem 5-i?

Copy and paste relevant sections from the manuscript (include quotes in quotation marks "like this" to indicate direct quotes from your manuscript), or elaborate on this item by providing additional information not in the ms, or briefly explain why the item is not applicable/relevant for your study

in the manuscript we refer to previous studies in which we report on development and validation of our tool

5-ii) Describe the history/development process

Describe the history/development process of the application and previous formative evaluations (e.g., focus groups, usability testing), as these will have an impact on adoption/use rates and help with interpreting results.

subitem not at all important ○ ○ ○ ◉ ○ essential

Does your paper address subitem 5-ii?

Copy and paste relevant sections from the manuscript (include quotes in quotation marks "like this" to indicate direct quotes from your manuscript), or elaborate on this item by providing additional information not in the ms, or briefly explain why the item is not applicable/relevant for your study

"in the manuscript we refer to previous studies in which we report on development and validation of our tool"

5-iii) Revisions and updating

Revisions and updating. Clearly mention the date and/or version number of the application/intervention (and comparator, if applicable) evaluated, or describe whether the intervention underwent major changes during the evaluation process, or whether the development and/or content was "frozen" during the trial. Describe dynamic components such as news feeds or changing content which may have an impact on the replicability of the intervention (for unexpected events see item 3b).

subitem not at all important ◉ ○ ○ ○ ○ essential

Does your paper address subitem 5-iii?

Copy and paste relevant sections from the manuscript (include quotes in quotation marks "like this" to indicate direct quotes from your manuscript), or elaborate on this item by providing additional information not in the ms, or briefly explain why the item is not applicable/relevant for your study

not applicable yet

5-iv) Quality assurance methods

Provide information on quality assurance methods to ensure accuracy and quality of information provided [1], if applicable.

subitem not at all important ○ ○ ○ ◉ ○ essential

Does your paper address subitem 5-iv?

Copy and paste relevant sections from the manuscript (include quotes in quotation marks "like this" to indicate direct quotes from your manuscript), or elaborate on this item by providing additional information not in the ms, or briefly explain why the item is not applicable/relevant for your study

"in the manuscript we refer to previous studies in which we report on development and validation of our tool"

5-v) Ensure replicability by publishing the source code, and/or providing screenshots/screen-capture video, and/or providing flowcharts of the algorithms used

Ensure replicability by publishing the source code, and/or providing screenshots/screen-capture video, and/or providing flowcharts of the algorithms used. Replicability (i.e., other researchers should in principle be able to replicate the study) is a hallmark of scientific reporting.

subitem not at all important ○ ○ ○ ◉ ○ essential

Does your paper address subitem 5-v?

Copy and paste relevant sections from the manuscript (include quotes in quotation marks "like this" to indicate direct quotes from your manuscript), or elaborate on this item by providing additional information not in the ms, or briefly explain why the item is not applicable/relevant for your study

in the main paper we refer to previous work in which algorithms are presented.

5-vi) Digital preservation

Digital preservation: Provide the URL of the application, but as the intervention is likely to change or disappear over the course of the years; also make sure the intervention is archived (Internet Archive, webcitation.org, and/or publishing the source code or screenshots/videos alongside the article). As pages behind login screens cannot be archived, consider creating demo pages which are accessible without login.

subitem not at all important ◉ ○ ○ ○ ○ essential

Does your paper address subitem 5-vi?

Copy and paste relevant sections from the manuscript (include quotes in quotation marks "like this" to indicate direct quotes from your manuscript), or elaborate on this item by providing additional information not in the ms, or briefly explain why the item is not applicable/relevant for your study

not applicable

5-vii) Access

Access: Describe how participants accessed the application, in what setting/context, if they had to pay (or were paid) or not, whether they had to be a member of specific group. If known, describe how participants obtained "access to the platform and Internet" [1]. To ensure access for editors/reviewers/readers, consider to provide a "backdoor" login account or demo mode for reviewers/readers to explore the application (also important for archiving purposes, see vi).

subitem not at all important ○ ○ ○ ◉ ○ essential

Does your paper address subitem 5-vii? *

Copy and paste relevant sections from the manuscript (include quotes in quotation marks "like this" to indicate direct quotes from your manuscript), or elaborate on this item by providing additional information not in the ms, or briefly explain why the item is not applicable/relevant for your study

" Before the trial started participants in the intervention group were instructed to use the system daily for two weeks in order to get familiarised with the application, smartphone, spirometer, pulse-oximeter, and forehead thermometer. Data were sent to a secured web-based interface and were monitored by the research team to make sure participants practiced sufficiently. After this 2-week run-in period, the nurses evaluated patients' use of the system, including the physiological measurements. Reference values for each patient's FEV1 and peripheral oxygen saturation were set. Then, the 12-month follow-up period started. Patients were instructed to use the tool every time they experienced or had any doubts about any change in symptoms or disease burden. At three months follow-up, patients were invited by their nurse to evaluate the use of the mHealth tool. Only then the nurses received from the research team the patients' entries to enable tailoring of feedback on self-management behaviour. "

5-viii) Mode of delivery, features/functionalities/components of the intervention and comparator, and the theoretical framework

Describe mode of delivery, features/functionalities/components of the intervention and comparator, and the theoretical framework [6] used to design them (instructional strategy [1], behaviour change techniques, persuasive features, etc., see e.g., [7, 8] for terminology). This includes an in-depth description of the content (including where it is coming from and who developed it) [1]," whether [and how] it is tailored to individual circumstances and allows users to track their progress and receive feedback" [6]. This also includes a description of communication delivery channels and – if computer-mediated communication is a component – whether communication was synchronous or asynchronous [6]. It also includes information on presentation strategies [1], including page design principles, average amount of text on pages, presence of hyperlinks to other resources, etc. [1].

subitem not at all important ○ ○ ○ ◉ ○ essential

Does your paper address subitem 5-viii? *

Copy and paste relevant sections from the manuscript (include quotes in quotation marks "like this" to indicate direct quotes from your manuscript), or elaborate on this item by providing additional information not in the ms, or briefly explain why the item is not applicable/relevant for your study

"The tool consisted of a smartphone (provided by the research team), a pulse-oximeter (CMS50D, Contec Medical Systems, P.R. China), a spirometer (PiKo-1 monitor, nSpire, United Kingdom) and a forehead thermometer (FTN, Medisana AG, Germany). Patients answered 12 yes-or-no questions concerning changes in symptoms, physical limitations and emotions by touch screen on the smartphone, complemented by measurements of the pulse-oximeter, spirometer and forehead thermometer (see Appendix 1).[17] All questions had to be answered to proceed. Based on a built-in Bayesian network decision model, the mHealth tool then provided one or more of the following advices; a) increase your bronchodilator use (including a personalised medication instruction), b) use your breathing techniques, c) use your coughing techniques, d) be thoughtful of how you distribute your energy during the day, e) contact your healthcare professional today, f) measure again tomorrow. Completing the questions and measurements took approximately 5 minutes. The mHealth tool has been developed in close collaboration with COPD patients and healthcare professionals [17] and has shown high sensitivity and specificity.[18] "

5-ix) Describe use parameters

Describe use parameters (e.g., intended "doses" and optimal timing for use). Clarify what instructions or recommendations were given to the user, e.g., regarding timing, frequency, heaviness of use, if any, or was the intervention used ad libitum.

subitem not at all important ○ ○ ○ ◉ ○ essential

Does your paper address subitem 5-ix?

Copy and paste relevant sections from the manuscript (include quotes in quotation marks "like this" to indicate direct quotes from your manuscript), or elaborate on this item by providing additional information not in the ms, or briefly explain why the item is not applicable/relevant for your study

"The tool consisted of a smartphone (provided by the research team), a pulse-oximeter (CMS50D, Contec Medical Systems, P.R. China), a spirometer (PiKo-1 monitor, nSpire, United Kingdom) and a forehead thermometer (FTN, Medisana AG, Germany). Patients answered 12 yes-or-no questions concerning changes in symptoms, physical limitations and emotions by touch screen on the smartphone, complemented by measurements of the pulse-oximeter, spirometer and forehead thermometer (see Appendix 1).[17] All questions had to be answered to proceed. Based on a built-in Bayesian network decision model, the mHealth tool then provided one or more of the following advices; a) increase your bronchodilator use (including a personalised medication instruction), b) use your breathing techniques, c) use your coughing techniques, d) be thoughtful of how you distribute your energy during the day, e) contact your healthcare professional today, f) measure again tomorrow. Completing the questions and measurements took approximately 5 minutes. The mHealth tool has been developed in close collaboration with COPD patients and healthcare professionals [17] and has shown high sensitivity and specificity.[18]"

5-x) Clarify the level of human involvement

Clarify the level of human involvement (care providers or health professionals, also technical assistance) in the e-intervention or as co-intervention (detail number and expertise of professionals involved, if any, as well as "type of assistance offered, the timing and frequency of the support, how it is initiated, and the medium by which the assistance is delivered". It may be necessary to distinguish between the level of human involvement required for the trial, and the level of human involvement required for a routine application outside of a RCT setting (discuss under item 21 – generalizability).

subitem not at all important ○ ○ ○ ◉ ○ essential

Does your paper address subitem 5-x?

Copy and paste relevant sections from the manuscript (include quotes in quotation marks "like this" to indicate direct quotes from your manuscript), or elaborate on this item by providing additional information not in the ms, or briefly explain why the item is not applicable/relevant for your study

"Participants in the intervention group were instructed to visit the nurse within two weeks after allocation for instructions on the use of the mHealth tool. The tool consisted of a smartphone (provided by the research team), a pulse-oximeter (CMS50D, Contec Medical Systems, P.R. China), a spirometer (PiKo-1 monitor, nSpire, United Kingdom) and a forehead thermometer (FTN, Medisana AG, Germany). Patients answered 12 yes-or-no questions concerning changes in symptoms, physical limitations and emotions by touch screen on the smartphone, complemented by measurements of the pulse-oximeter, spirometer and forehead thermometer (see Appendix 1).[17] All questions had to be answered to proceed. Based on a built-in Bayesian network decision model, the mHealth tool then provided one or more of the following advices; a) increase your bronchodilator use (including a personalised medication instruction), b) use your breathing techniques, c) use your coughing techniques, d) be thoughtful of how you distribute your energy during the day, e) contact your healthcare professional today, f) measure again tomorrow. Completing the questions and measurements took approximately 5 minutes. The mHealth tool has been developed in close collaboration with COPD patients and healthcare professionals [17] and has shown high sensitivity and specificity.[18]
Before the trial started participants in the intervention group were instructed to use the system daily for two weeks in order to get familiarised with the application, smartphone, spirometer, pulse-oximeter, and forehead thermometer. Data were sent to a secured web-based interface and were monitored by the research team to make sure participants practiced sufficiently. After this 2-week run-in period, the nurses evaluated patients' use of the system, including the physiological measurements. Reference values for each patient's FEV1 and peripheral oxygen saturation were set. Then, the 12-month follow-up period started. Patients were instructed to use the tool every time they experienced or had any doubts about any change in symptoms or disease burden. At three months follow-up, patients were invited by their nurse to evaluate the use of the mHealth tool. Only then the nurses received from the research team the patients' entries to enable tailoring of feedback on self-management behaviour.
"

5-xi) Report any prompts/reminders used

Report any prompts/reminders used: Clarify if there were prompts (letters, emails, phone calls, SMS) to use the application, what triggered them, frequency etc. It may be necessary to distinguish between the level of prompts/reminders required for the trial, and the level of prompts/reminders for a routine application outside of a RCT setting (discuss under item 21 – generalizability).

subitem not at all important ○ ○ ○ ◉ ○ essential

Does your paper address subitem 5-xi? *

Copy and paste relevant sections from the manuscript (include quotes in quotation marks "like this" to indicate direct quotes from your manuscript), or elaborate on this item by providing additional information not in the ms, or briefly explain why the item is not applicable/relevant for your study

"At three months follow-up, patients were invited by their nurse to evaluate the use of the mHealth tool. Only then the nurses received from the research team the patients' entries to enable tailoring of feedback on self-management behaviour. "

5-xii) Describe any co-interventions (incl. training/support)

Describe any co-interventions (incl. training/support): Clearly state any interventions that are provided in addition to the targeted eHealth intervention, as ehealth intervention may not be designed as stand-alone intervention. This includes training sessions and support [1]. It may be necessary to distinguish between the level of training required for the trial, and the level of training for a routine application outside of a RCT setting (discuss under item 21 – generalizability.

subitem not at all important ○ ○ ○ ◉ ○ essential

Does your paper address subitem 5-xii? *

Copy and paste relevant sections from the manuscript (include quotes in quotation marks "like this" to indicate direct quotes from your manuscript), or elaborate on this item by providing additional information not in the ms, or briefly explain why the item is not applicable/relevant for your study

"Prior to randomization and after signing written informed consent participants received a 20-minute educational session based on the Dutch version of the Living Well with COPD self-management program provided by the nurse in groups of 4 to 10 participants, to establish a homogeneous baseline in exacerbation self-management knowledge.[12] "
"Before the trial started participants in the intervention group were instructed to use the system daily for two weeks in order to get familiarised with the application, smartphone, spirometer, pulse-oximeter, and forehead thermometer. "

6a) Completely defined pre-specified primary and secondary outcome measures, including how and when they were assessed

Does your paper address CONSORT subitem 6a? *

Copy and paste relevant sections from the manuscript (include quotes in quotation marks "like this" to indicate direct quotes from your manuscript), or elaborate on this item by providing additional information not in the ms, or briefly explain why the item is not applicable/relevant for your study

"Outcomes and follow-up
Our primary outcome was the difference in number of exacerbation-free weeks between the intervention and control groups. An exacerbation-free week was defined as a week in which there had not been episodes of two or more consecutive days with worsening of two major symptoms (i.e. dyspnoea, sputum purulence, sputum amount) or one major and one or more minor symptoms (i.e. colds, wheeze, sore throat, and cough).[21] Symptom changes were assessed by means of the Telephonic Exacerbation Assessment System (TEXAS), an automated telephone call system that contacted participants weekly on the day and time of their preference.[22] TEXAS consisted of closed questions regarding changes in respiratory symptoms, use of healthcare resources and use of respiratory medication in the week prior to the call and its validity has been demonstrated previously.[22] Due to discontinuation of the contract with the provider of TEXAS, the last 19 participants in the trial received a weekly online questionnaire containing the same questions as with TEXAS. These participants used both measuring tools during two weeks before stopping with TEXAS, which enabled us to compare data entry from TEXAS with the online survey tool. We found no differences in data entry.
Secondary outcomes included:

- Exacerbation-related outcomes, i.e. the number of unscheduled healthcare contacts, the number of exacerbations treated with antibiotics and/or prednisolone, and the number of exacerbation-related hospital admissions, all retrieved from patients' medical records, and the number of symptom-based exacerbations as assessed with TEXAS.
- Exacerbation-related self-management behaviour, measured with TEXAS or the online questionnaire, and defined as taken one or more of the following three actions during symptom-based exacerbations: (i) contacting the healthcare professional , (ii) starting a course of prednisolone and/or antibiotics, or (iii) maximising bronchodilator use. We also assessed the time between the date of exacerbation onset and the date of one of these three actions, defining actions taken within 3 days of exacerbation onset as adherence to the instructions.
- Exacerbation-related self-efficacy, measured with an exacerbation-related self-efficacy scale containing 5 questions. This questionnaire was created for the purpose of this study, since to our knowledge no questionnaire existed that measured exacerbation-related self-efficacy. Reliability analyses showed a Cronbach's alpha of 0.69 at baseline and 0.81 at follow-up.
- Health status, measured with (i) the Nijmegen Clinical Screening Instrument (NCSI), which is a battery of instruments measuring eight sub-domains of health status –subjective symptoms, dyspnoea emotions, fatigue, behavioural impairment, subjective impairment, general quality of life (QoL), health-related QoL and satisfaction with relationships [23], (ii) the Clinical COPD Questionnaire (CCQ), which measures 3 sub-domains, i.e. symptoms, functional status and mental status, resulting in a total score (24); and (iii) the EuroQol-5 dimensions (EQ-5d),[24] which measures health-related quality of life with a total score based on weighted scores on mobility, self-care, usual activities, pain/discomfort and anxiety/depression, as well as a vertical VAS scale varying between 0 and 100.

At the start and at 12 months, data were gathered on exacerbation history, self-efficacy and health status. CCQ and EQ-5d were also completed at three, six, and nine months follow-up.

At 12 months, information on healthcare utilization, lung function, respiratory medication use, and comorbid conditions were extracted from the participants' medical records. Also, all participants were asked to evaluate the supportive function of either the mHealth tool or the paper action plan and participants of the intervention group were asked to complete the System Usability Scale (SUS).[25] The SUS contains 10 questions on system usability, which are calculated into one total score between 0 and 100. SUS scores < 68 are considered as low, ≥ 68 and ≤ 80.3 as good, and > 80.3 as excellent. "

6a-i) Online questionnaires: describe if they were validated for online use and apply CHERRIES items to describe how the questionnaires were designed/deployed

If outcomes were obtained through online questionnaires, describe if they were validated for online use and apply CHERRIES items to describe how the questionnaires were designed/deployed [9].

subitem not at all important ◉ ○ ○ ○ ○ essential

Does your paper address subitem 6a-i?

Copy and paste relevant sections from manuscript text

not relevant

6a-ii) Describe whether and how “use” (including intensity of use/dosage) was defined/measured/monitored

Describe whether and how “use” (including intensity of use/dosage) was defined/measured/monitored (logins, logfile analysis, etc.). Use/adoption metrics are important process outcomes that should be reported in any ehealth trial.

subitem not at all important ○ ○ ○ ◉ ○ essential

Does your paper address subitem 6a-ii?

Copy and paste relevant sections from manuscript text

"Also, all participants were asked to evaluate the supportive function of either the mHealth tool or the paper action plan and participants of the intervention group were asked to complete the System Usability Scale (SUS).[25] The SUS contains 10 questions on system usability, which are calculated into one total score between 0 and 100. SUS scores < 68 are considered as low, ≥ 68 and ≤ 80.3 as good, and > 80.3 as excellent. "

6a-iii) Describe whether, how, and when qualitative feedback from participants was obtained

Describe whether, how, and when qualitative feedback from participants was obtained (e.g., through emails, feedback forms, interviews, focus groups).

subitem not at all important ○ ○ ○ ◉ ○ essential

Does your paper address subitem 6a-iii?

Copy and paste relevant sections from manuscript text

"Also, all participants were asked to evaluate the supportive function of either the mHealth tool or the paper action plan and participants of the intervention group were asked to complete the System Usability Scale (SUS).[25] The SUS contains 10 questions on system usability, which are calculated into one total score between 0 and 100. SUS scores < 68 are considered as low, ≥ 68 and ≤ 80.3 as good, and > 80.3 as excellent. "

6b) Any changes to trial outcomes after the trial commenced, with reasons

Does your paper address CONSORT subitem 6b? *

Copy and paste relevant sections from the manuscript (include quotes in quotation marks "like this" to indicate direct quotes from your manuscript), or elaborate on this item by providing additional information not in the ms, or briefly explain why the item is not applicable/relevant for your study

not applicable

7a) How sample size was determined

NPT: When applicable, details of whether and how the clustering by care provides or centers was addressed

7a-i) Describe whether and how expected attrition was taken into account when calculating the sample size

Describe whether and how expected attrition was taken into account when calculating the sample size.

subitem not at all important ○ ○ ○ ◉ ○ essential

Does your paper address subitem 7a-i?

Copy and paste relevant sections from manuscript title (include quotes in quotation marks "like this" to indicate direct quotes from your manuscript), or elaborate on this item by providing additional information not in the ms, or briefly explain why the item is not applicable/relevant for your study

"Sample size calculation using analysis of variance showed that we needed 43 participants in each group for 80% power (α=0.05, two sided) to detect an increase of six exacerbation-free weeks per year and anticipating a drop-out rate of 20%. "

7b) When applicable, explanation of any interim analyses and stopping guidelines

Does your paper address CONSORT subitem 7b? *

Copy and paste relevant sections from the manuscript (include quotes in quotation marks "like this" to indicate direct quotes from your manuscript), or elaborate on this item by providing additional information not in the ms, or briefly explain why the item is not applicable/relevant for your study

not applicable

8a) Method used to generate the random allocation sequence

NPT: When applicable, how care providers were allocated to each trial group

Does your paper address CONSORT subitem 8a? *

Copy and paste relevant sections from the manuscript (include quotes in quotation marks "like this" to indicate direct quotes from your manuscript), or elaborate on this item by providing additional information not in the ms, or briefly explain why the item is not applicable/relevant for your study

"We used a computer generated two-block randomization procedure, stratifying for healthcare centre. Allocation order was determined by the order in which eligible patients responded to our invitation to participate (kept by the research assistant). Participants were assigned to one of the groups after signing informed consent, during the group meeting by the researcher (LB). "

8b) Type of randomisation; details of any restriction (such as blocking and block size)

Does your paper address CONSORT subitem 8b? *

Copy and paste relevant sections from the manuscript (include quotes in quotation marks "like this" to indicate direct quotes from your manuscript), or elaborate on this item by providing additional information not in the ms, or briefly explain why the item is not applicable/relevant for your study

"We used a computer generated two-block randomization procedure, stratifying for healthcare centre. Allocation order was determined by the order in which eligible patients responded to our invitation to participate (kept by the research assistant). Participants were assigned to one of the groups after signing informed consent, during the group meeting by the researcher (LB). "

9) Mechanism used to implement the random allocation sequence (such as sequentially numbered containers), describing any steps taken to conceal the sequence until interventions were assigned

Does your paper address CONSORT subitem 9? *

Copy and paste relevant sections from the manuscript (include quotes in quotation marks "like this" to indicate direct quotes from your manuscript), or elaborate on this item by providing additional information not in the ms, or briefly explain why the item is not applicable/relevant for your study

" We used a computer generated two-block randomization procedure, stratifying for healthcare centre. Allocation order was determined by the order in which eligible patients responded to our invitation to participate (kept by the research assistant). Participants were assigned to one of the groups after signing informed consent, during the group meeting by the researcher (LB). "

10) Who generated the random allocation sequence, who enrolled participants, and who assigned participants to interventions

Does your paper address CONSORT subitem 10? *

Copy and paste relevant sections from the manuscript (include quotes in quotation marks "like this" to indicate direct quotes from your manuscript), or elaborate on this item by providing additional information not in the ms, or briefly explain why the item is not applicable/relevant for your study

"We used a computer generated two-block randomization procedure, stratifying for healthcare centre. Allocation order was determined by the order in which eligible patients responded to our invitation to participate (kept by the research assistant). Participants were assigned to one of the groups after signing informed consent, during the group meeting by the researcher (LB). "

11a) If done, who was blinded after assignment to interventions (for example, participants, care providers, those assessing outcomes) and how

NPT: Whether or not administering co-interventions were blinded to group assignment

11a-i) Specify who was blinded, and who wasn't

Specify who was blinded, and who wasn't. Usually, in web-based trials it is not possible to blind the participants [1, 3] (this should be clearly acknowledged), but it may be possible to blind outcome assessors, those doing data analysis or those administering co-interventions (if any).

subitem not at all important ○ ○ ○ ◉ ○ essential

Does your paper address subitem 11a-i? *

Copy and paste relevant sections from the manuscript (include quotes in quotation marks "like this" to indicate direct quotes from your manuscript), or elaborate on this item by providing additional information not in the ms, or briefly explain why the item is not applicable/relevant for your study

"Due to the type of intervention, patients and healthcare professionals could not be blinded for group assignment. Also, the research team could not be blinded, since it was responsible for the personalisation and technical support of the mHealth tool. The study statistician (RA), who was responsible for analysing the data, was blinded for study assignment of the participants until the analyses had been finished."

11a-ii) Discuss e.g., whether participants knew which intervention was the “intervention of interest” and which one was the “comparator”

Informed consent procedures (4a-ii) can create biases and certain expectations - discuss e.g., whether participants knew which intervention was the “intervention of interest” and which one was the “comparator”.

subitem not at all important ○ ○ ○ ◉ ○ essential

Does your paper address subitem 11a-ii?

Copy and paste relevant sections from the manuscript (include quotes in quotation marks "like this" to indicate direct quotes from your manuscript), or elaborate on this item by providing additional information not in the ms, or briefly explain why the item is not applicable/relevant for your study

"Due to the type of intervention, patients and healthcare professionals could not be blinded for group assignment. Also, the research team could not be blinded, since it was responsible for the personalisation and technical support of the mHealth tool. The study statistician (RA), who was responsible for analysing the data, was blinded for study assignment of the participants until the analyses had been finished. "

11b) If relevant, description of the similarity of interventions

(this item is usually not relevant for ehealth trials as it refers to similarity of a placebo or sham intervention to a active medication/intervention)

Does your paper address CONSORT subitem 11b? *

Copy and paste relevant sections from the manuscript (include quotes in quotation marks "like this" to indicate direct quotes from your manuscript), or elaborate on this item by providing additional information not in the ms, or briefly explain why the item is not applicable/relevant for your study

not relevant

12a) Statistical methods used to compare groups for primary and secondary outcomes

NPT: When applicable, details of whether and how the clustering by care providers or centers was addressed

Does your paper address CONSORT subitem 12a? *

Copy and paste relevant sections from the manuscript (include quotes in quotation marks "like this" to indicate direct quotes from your manuscript), or elaborate on this item by providing additional information not in the ms, or briefly explain why the item is not applicable/relevant for your study

"Separate multilevel logistic regression analyses were performed, taking into account the clustering effect of exacerbations within patients"

12a-i) Imputation techniques to deal with attrition / missing values

Imputation techniques to deal with attrition / missing values: Not all participants will use the intervention/comparator as intended and attrition is typically high in ehealth trials. Specify how participants who did not use the application or dropped out from the trial were treated in the statistical analysis (a complete case analysis is strongly discouraged, and simple imputation techniques such as LOCF may also be problematic [4]).

subitem not at all important ○ ○ ○ ◉ ○ essential

Does your paper address subitem 12a-i? *

Copy and paste relevant sections from the manuscript (include quotes in quotation marks "like this" to indicate direct quotes from your manuscript), or elaborate on this item by providing additional information not in the ms, or briefly explain why the item is not applicable/relevant for your study

"Missing data were not imputed."

12b) Methods for additional analyses, such as subgroup analyses and adjusted analyses

Does your paper address CONSORT subitem 12b? *

Copy and paste relevant sections from the manuscript (include quotes in quotation marks "like this" to indicate direct quotes from your manuscript), or elaborate on this item by providing additional information not in the ms, or briefly explain why the item is not applicable/relevant for your study

"Negative binomial regression analyses, controlling for follow-up time per participant, age and gender, were used to analyse our primary outcome, i.e. the number of exacerbation-free weeks, as well as the number of unscheduled healthcare contacts, self-reported exacerbations, exacerbations treated with antibiotics and/or prednisolone, and exacerbation-related hospital admissions. To test the effect of the mHealth tool on the rate of symptom-based exacerbations and self-management behaviour, we extracted exacerbation episodes from the TEXAS database. Each new episode was preceded by at least two exacerbation-free weeks or two weeks with missing data.[22] To assess patient delay in taking action when an exacerbation was imminent, the numbers of days were calculated between the date of exacerbation onset and the following actions: a) the first date of contact with a healthcare professional, b) starting date of course of prednisolone and/or antibiotics, or c) date of increase of bronchodilators. We categorised these variables into two groups: <3 days (according to instructions), and ≥3 days. Separate multilevel logistic regression analyses were performed, taking into account the clustering effect of exacerbations within patients, and controlling for age and gender, to examine a) whether the mHealth tool led to higher percentages of self-management actions in case of an exacerbation compared to the paper action plan, and b) whether these actions were more often taken timely by patients in the intervention group compared to the control group.
"

X26) REB/IRB Approval and Ethical Considerations [recommended as subheading under "Methods"] (not a CONSORT item)

X26-i) Comment on ethics committee approval

subitem not at all important ○ ○ ○ ◉ ○ essential

Does your paper address subitem X26-i?

Copy and paste relevant sections from the manuscript (include quotes in quotation marks "like this" to indicate direct quotes from your manuscript), or elaborate on this item by providing additional information not in the ms, or briefly explain why the item is not applicable/relevant for your study

"This study has been registered at Clinicaltrials.gov (Identifier: NCT02553096) and has been approved by the Medical Ethics Review Board, region Arnhem-Nijmegen, the Netherlands (file 2014-1270)."

x26-ii) Outline informed consent procedures

Outline informed consent procedures e.g., if consent was obtained offline or online (how? Checkbox, etc.?), and what information was provided (see 4a-ii). See [6] for some items to be included in informed consent documents.

subitem not at all important ◉ ○ ○ ○ ○ essential

Does your paper address subitem X26-ii?

Copy and paste relevant sections from the manuscript (include quotes in quotation marks "like this" to indicate direct quotes from your manuscript), or elaborate on this item by providing additional information not in the ms, or briefly explain why the item is not applicable/relevant for your study

not relevant

X26-iii) Safety and security procedures

Safety and security procedures, incl. privacy considerations, and any steps taken to reduce the likelihood or detection of harm (e.g., education and training, availability of a hotline)

subitem not at all important ○ ○ ◉ ○ ○ essential

Does your paper address subitem X26-iii?

Copy and paste relevant sections from the manuscript (include quotes in quotation marks "like this" to indicate direct quotes from your manuscript), or elaborate on this item by providing additional information not in the ms, or briefly explain why the item is not applicable/relevant for your study

"11 COPD-related hospital admissions (6 in the intervention group and 5 in the control group) were reported as serious adverse events to the medical ethics review board. "

RESULTS

13a) For each group, the numbers of participants who were randomly assigned, received intended treatment, and were analysed for the primary outcome

NPT: The number of care providers or centers performing the intervention in each group and the number of patients treated by each care provider in each center

Does your paper address CONSORT subitem 13a? *

Copy and paste relevant sections from the manuscript (include quotes in quotation marks "like this" to indicate direct quotes from your manuscript), or elaborate on this item by providing additional information not in the ms, or briefly explain why the item is not applicable/relevant for your study

yes

13b) For each group, losses and exclusions after randomisation, together with reasons

Does your paper address CONSORT subitem 13b? (NOTE: Preferably, this is shown in a CONSORT flow diagram) *

Copy and paste relevant sections from the manuscript (include quotes in quotation marks "like this" to indicate direct quotes from your manuscript), or elaborate on this item by providing additional information not in the ms, or briefly explain why the item is not applicable/relevant for your study

yes, this is shown in flow diagram

13b-i) Attrition diagram

Strongly recommended: An attrition diagram (e.g., proportion of participants still logging in or using the intervention/comparator in each group plotted over time, similar to a survival curve) or other figures or tables demonstrating usage/dose/engagement.

subitem not at all important ○ ○ ◉ ○ ○ essential

Does your paper address subitem 13b-i?

Copy and paste relevant sections from the manuscript or cite the figure number if applicable (include quotes in quotation marks "like this" to indicate direct quotes from your manuscript), or elaborate on this item by providing additional information not in the ms, or briefly explain why the item is not applicable/relevant for your study

attrition diagram has not been made, data about actual use of mHealth tool or comparator is not available

14a) Dates defining the periods of recruitment and follow-up

Does your paper address CONSORT subitem 14a? *

Copy and paste relevant sections from the manuscript (include quotes in quotation marks "like this" to indicate direct quotes from your manuscript), or elaborate on this item by providing additional information not in the ms, or briefly explain why the item is not applicable/relevant for your study

"Patients were recruited between June 2015 and July 2016 at the pulmonary outpatient clinics of three Dutch hospitals and nine general practices in the city of Nijmegen and surroundings, the Netherlands. "

14a-i) Indicate if critical “secular events” fell into the study period

Indicate if critical “secular events” fell into the study period, e.g., significant changes in Internet resources available or "changes in computer hardware or Internet delivery resources"

subitem not at all important	◉	○	○	○	○	essential

Does your paper address subitem 14a-i?

Copy and paste relevant sections from the manuscript (include quotes in quotation marks "like this" to indicate direct quotes from your manuscript), or elaborate on this item by providing additional information not in the ms, or briefly explain why the item is not applicable/relevant for your study

not relevant

14b) Why the trial ended or was stopped (early)

Does your paper address CONSORT subitem 14b? *

Copy and paste relevant sections from the manuscript (include quotes in quotation marks "like this" to indicate direct quotes from your manuscript), or elaborate on this item by providing additional information not in the ms, or briefly explain why the item is not applicable/relevant for your study

not applicable

15) A table showing baseline demographic and clinical characteristics for each group

NPT: When applicable, a description of care providers (case volume, qualification, expertise, etc.) and centers (volume) in each group

Does your paper address CONSORT subitem 15? *

Copy and paste relevant sections from the manuscript (include quotes in quotation marks "like this" to indicate direct quotes from your manuscript), or elaborate on this item by providing additional information not in the ms, or briefly explain why the item is not applicable/relevant for your study

yes

15-i) Report demographics associated with digital divide issues

In ehealth trials it is particularly important to report demographics associated with digital divide issues, such as age, education, gender, social-economic status, computer/Internet/ehealth literacy of the participants, if known.

subitem not at all important	◉	○	○	○	○	essential

Does your paper address subitem 15-i? *

Copy and paste relevant sections from the manuscript (include quotes in quotation marks "like this" to indicate direct quotes from your manuscript), or elaborate on this item by providing additional information not in the ms, or briefly explain why the item is not applicable/relevant for your study

not known

16) For each group, number of participants (denominator) included in each analysis and whether the analysis was by original assigned groups

16-i) Report multiple “denominators” and provide definitions

Report multiple “denominators” and provide definitions: Report N's (and effect sizes) "across a range of study participation [and use] thresholds" [1], e.g., N exposed, N consented, N used more than x times, N used more than y weeks, N participants “used” the intervention/comparator at specific pre-defined time points of interest (in absolute and relative numbers per group). Always clearly define “use” of the intervention.

subitem not at all important ○ ○ ○ ◉ ○ essential

Does your paper address subitem 16-i? *

Copy and paste relevant sections from the manuscript (include quotes in quotation marks "like this" to indicate direct quotes from your manuscript), or elaborate on this item by providing additional information not in the ms, or briefly explain why the item is not applicable/relevant for your study

yes

16-ii) Primary analysis should be intent-to-treat

Primary analysis should be intent-to-treat, secondary analyses could include comparing only “users”, with the appropriate caveats that this is no longer a randomized sample (see 18-i).

subitem not at all important ○ ○ ○ ◉ ○ essential

Does your paper address subitem 16-ii?

Copy and paste relevant sections from the manuscript (include quotes in quotation marks "like this" to indicate direct quotes from your manuscript), or elaborate on this item by providing additional information not in the ms, or briefly explain why the item is not applicable/relevant for your study

yes

17a) For each primary and secondary outcome, results for each group, and the estimated effect size and its precision (such as 95% confidence interval)

Does your paper address CONSORT subitem 17a? *

Copy and paste relevant sections from the manuscript (include quotes in quotation marks "like this" to indicate direct quotes from your manuscript), or elaborate on this item by providing additional information not in the ms, or briefly explain why the item is not applicable/relevant for your study

yes

17a-i) Presentation of process outcomes such as metrics of use and intensity of use

In addition to primary/secondary (clinical) outcomes, the presentation of process outcomes such as metrics of use and intensity of use (dose, exposure) and their operational definitions is critical. This does not only refer to metrics of attrition (13-b) (often a binary variable), but also to more continuous exposure metrics such as “average session length”. These must be accompanied by a technical description how a metric like a “session” is defined (e.g., timeout after idle time) [1] (report under item 6a).

subitem not at all important ○ ○ ○ ◉ ○ essential

Does your paper address subitem 17a-i?

Copy and paste relevant sections from the manuscript (include quotes in quotation marks "like this" to indicate direct quotes from your manuscript), or elaborate on this item by providing additional information not in the ms, or briefly explain why the item is not applicable/relevant for your study

"Mean duration of follow-up was 48.1 (SD 11.7) weeks, "

17b) For binary outcomes, presentation of both absolute and relative effect sizes is recommended

Does your paper address CONSORT subitem 17b? *

Copy and paste relevant sections from the manuscript (include quotes in quotation marks "like this" to indicate direct quotes from your manuscript), or elaborate on this item by providing additional information not in the ms, or briefly explain why the item is not applicable/relevant for your study

yes

18) Results of any other analyses performed, including subgroup analyses and adjusted analyses, distinguishing pre-specified from exploratory

Does your paper address CONSORT subitem 18? *

Copy and paste relevant sections from the manuscript (include quotes in quotation marks "like this" to indicate direct quotes from your manuscript), or elaborate on this item by providing additional information not in the ms, or briefly explain why the item is not applicable/relevant for your study

yes

18-i) Subgroup analysis of comparing only users

A subgroup analysis of comparing only users is not uncommon in ehealth trials, but if done, it must be stressed that this is a self-selected sample and no longer an unbiased sample from a randomized trial (see 16-iii).

subitem not at all important ◉ ○ ○ ○ ○ essential

Does your paper address subitem 18-i?

Copy and paste relevant sections from the manuscript (include quotes in quotation marks "like this" to indicate direct quotes from your manuscript), or elaborate on this item by providing additional information not in the ms, or briefly explain why the item is not applicable/relevant for your study

we did not perform subgroup analyses

19) All important harms or unintended effects in each group

(for specific guidance see CONSORT for harms)

Does your paper address CONSORT subitem 19? *

Copy and paste relevant sections from the manuscript (include quotes in quotation marks "like this" to indicate direct quotes from your manuscript), or elaborate on this item by providing additional information not in the ms, or briefly explain why the item is not applicable/relevant for your study

yes

19-i) Include privacy breaches, technical problems

Include privacy breaches, technical problems. This does not only include physical "harm" to participants, but also incidents such as perceived or real privacy breaches [1], technical problems, and other unexpected/unintended incidents. "Unintended effects" also includes unintended positive effects [2].

subitem not at all important ◉ ○ ○ ○ ○ essential

Does your paper address subitem 19-i?

Copy and paste relevant sections from the manuscript (include quotes in quotation marks "like this" to indicate direct quotes from your manuscript), or elaborate on this item by providing additional information not in the ms, or briefly explain why the item is not applicable/relevant for your study

not applicable

19-ii) Include qualitative feedback from participants or observations from staff/researchers

Include qualitative feedback from participants or observations from staff/researchers, if available, on strengths and shortcomings of the application, especially if they point to unintended/unexpected effects or uses. This includes (if available) reasons for why people did or did not use the application as intended by the developers.

subitem not at all important ○ ○ ○ ◉ ○ essential

Does your paper address subitem 19-ii?

Copy and paste relevant sections from the manuscript (include quotes in quotation marks "like this" to indicate direct quotes from your manuscript), or elaborate on this item by providing additional information not in the ms, or briefly explain why the item is not applicable/relevant for your study

"all participants were asked to evaluate the supportive function of either the mHealth tool or the paper action plan and participants of the intervention group were asked to complete the System Usability Scale (SUS).[25] The SUS contains 10 questions on system usability, which are calculated into one total score between 0 and 100. SUS scores < 68 are considered as low, ≥ 68 and ≤ 80.3 as good, and > 80.3 as excellent. "

DISCUSSION

22) Interpretation consistent with results, balancing benefits and harms, and considering other relevant evidence

NPT: In addition, take into account the choice of the comparator, lack of or partial blinding, and unequal expertise of care providers or centers in each group

22-i) Restate study questions and summarize the answers suggested by the data, starting with primary outcomes and process outcomes (use)

Restate study questions and summarize the answers suggested by the data, starting with primary outcomes and process outcomes (use).

subitem not at all important ○ ○ ○ ◉ ○ essential

Does your paper address subitem 22-i? *

Copy and paste relevant sections from the manuscript (include quotes in quotation marks "like this" to indicate direct quotes from your manuscript), or elaborate on this item by providing additional information not in the ms, or briefly explain why the item is not applicable/relevant for your study

"In this study we examined the clinical effects of a smart mHealth tool to support COPD patients in the detection and treatment of exacerbations without the interference of a healthcare professional. Our primary hypothesis, that the use of mHealth would lead to more weeks without exacerbations than care as usual, i.e. the use of a paper action plan, was not confirmed. Also, we did not find differences in exacerbation frequency, healthcare utilisation or self-management behaviour between patients who used the mHealth tool and patients who used the paper action plan. Furthermore, patients using the tool did not report higher exacerbation-related self-efficacy or better health status than patients using a paper action plan. Patients evaluated the usability of the mHealth tool as good and considered it as more supportive than the action plan. "

22-ii) Highlight unanswered new questions, suggest future research

Highlight unanswered new questions, suggest future research.

subitem not at all important ○ ○ ○ ◉ ○ essential

Does your paper address subitem 22-ii?

Copy and paste relevant sections from the manuscript (include quotes in quotation marks "like this" to indicate direct quotes from your manuscript), or elaborate on this item by providing additional information not in the ms, or briefly explain why the item is not applicable/relevant for your study

"Future research
Patients using the mHealth tool evaluated it as usable and more supportive than patients using a written action plan. Future research should focus on patients who are specifically interested in using digital tools in their daily life, as these patients may have greater benefit from them.[28] This would better fit the goals of patient-centred care, where this software application can be used to tailor COPD exacerbation self-management, depending on the patient’s preference. "

20) Trial limitations, addressing sources of potential bias, imprecision, and, if relevant, multiplicity of analyses

20-i) Typical limitations in ehealth trials

Typical limitations in ehealth trials: Participants in ehealth trials are rarely blinded. Ehealth trials often look at a multiplicity of outcomes, increasing risk for a Type I error. Discuss biases due to non-use of the intervention/usability issues, biases through informed consent procedures, unexpected events.

subitem not at all important ○ ○ ○ ◉ ○ essential

Does your paper address subitem 20-i? *

Copy and paste relevant sections from the manuscript (include quotes in quotation marks "like this" to indicate direct quotes from your manuscript), or elaborate on this item by providing additional information not in the ms, or briefly explain why the item is not applicable/relevant for your study

yes

21) Generalisability (external validity, applicability) of the trial findings

NPT: External validity of the trial findings according to the intervention, comparators, patients, and care providers or centers involved in the trial

21-i) Generalizability to other populations

Generalizability to other populations: In particular, discuss generalizability to a general Internet population, outside of a RCT setting, and general patient population, including applicability of the study results for other organizations

subitem not at all important ○ ○ ○ ◉ ○ essential

Does your paper address subitem 21-i?

Copy and paste relevant sections from the manuscript (include quotes in quotation marks "like this" to indicate direct quotes from your manuscript), or elaborate on this item by providing additional information not in the ms, or briefly explain why the item is not applicable/relevant for your study

yes

21-ii) Discuss if there were elements in the RCT that would be different in a routine application setting

Discuss if there were elements in the RCT that would be different in a routine application setting (e.g., prompts/reminders, more human involvement, training sessions or other co-interventions) and what impact the omission of these elements could have on use, adoption, or outcomes if the intervention is applied outside of a RCT setting.

subitem not at all important ○ ○ ○ ◉ ○ essential

Does your paper address subitem 21-ii?

Copy and paste relevant sections from the manuscript (include quotes in quotation marks "like this" to indicate direct quotes from your manuscript), or elaborate on this item by providing additional information not in the ms, or briefly explain why the item is not applicable/relevant for your study

"Additionally, we chose to provide our control group with a paper action plan, according to the recommendations in the current national COPD guideline.[19] However, many general practitioners and chest physicians in the Netherlands have not (yet) integrated the use of paper action plans in their daily practice. Our choice might have upgraded self-management knowledge and skills of all participants, which may have reduced the room for improvement by mHealth and may have diluted potential differences in our outcomes, compared to a situation with 'real' (partially insufficient) usual care. "

OTHER INFORMATION

23) Registration number and name of trial registry

Does your paper address CONSORT subitem 23? *

Copy and paste relevant sections from the manuscript (include quotes in quotation marks "like this" to indicate direct quotes from your manuscript), or elaborate on this item by providing additional information not in the ms, or briefly explain why the item is not applicable/relevant for your study

This study has been registered at Clinicaltrials.gov (Identifier: NCT02553096)

24) Where the full trial protocol can be accessed, if available

Does your paper address CONSORT subitem 24? *

Cite a Multimedia Appendix, other reference, or copy and paste relevant sections from the manuscript (include quotes in quotation marks "like this" to indicate direct quotes from your manuscript), or elaborate on this item by providing additional information not in the ms, or briefly explain why the item is not applicable/relevant for your study

trial protocol can be checked at Clinicaltrials.gov

25) Sources of funding and other support (such as supply of drugs), role of funders

Does your paper address CONSORT subitem 25? *

Copy and paste relevant sections from the manuscript (include quotes in quotation marks "like this" to indicate direct quotes from your manuscript), or elaborate on this item by providing additional information not in the ms, or briefly explain why the item is not applicable/relevant for your study

not relevant

X27) Conflicts of Interest (not a CONSORT item)

X27-i) State the relation of the study team towards the system being evaluated

In addition to the usual declaration of interests (financial or otherwise), also state the relation of the study team towards the system being evaluated, i.e., state if the authors/evaluators are distinct from or identical with the developers/sponsors of the intervention.

subitem not at all important ○ ○ ○ ◉ ○ essential

Does your paper address subitem X27-i?

Copy and paste relevant sections from the manuscript (include quotes in quotation marks "like this" to indicate direct quotes from your manuscript), or elaborate on this item by providing additional information not in the ms, or briefly explain why the item is not applicable/relevant for your study

"None of the authors received any support from any company for the submitted work. All authors have completed the Unified Competing Interest form at www.icmje.org/coi_disclosure.pdf (available on request from the corresponding author) and declare: LB, MvdH, RA, YH, JV, and WA have nothing to disclose. PL has a patent 20140206949 issued to Petrus Lucas, EB received personal fees from Boehringer Ingelheim and GlaxoSmithKline, outside the submitted work, TS received a grant from GlaxoSmithKline and personal fees from Boehringer Ingelheim, outside the submitted work. "

About the CONSORT EHEALTH checklist

As a result of using this checklist, did you make changes in your manuscript? *

- ○ yes, major changes
- ○ yes, minor changes
- ◉ no

What were the most important changes you made as a result of using this checklist?

none

How much time did you spend on going through the checklist INCLUDING making changes in your manuscript *

60 minutes

As a result of using this checklist, do you think your manuscript has improved? *

- ◉ yes
- ○ no
- ○ Anders:

Would you like to become involved in the CONSORT EHEALTH group?

This would involve for example becoming involved in participating in a workshop and writing an "Explanation and Elaboration" document

- (x) yes
- ( ) no
- ( ) Anders:

Any other comments or questions on CONSORT EHEALTH

Jouw antwoord

STOP - Save this form as PDF before you click submit

To generate a record that you filled in this form, we recommend to generate a PDF of this page (on a Mac, simply select "print" and then select "print as PDF") before you submit it.

When you submit your (revised) paper to JMIR, please upload the PDF as supplementary file.

Don't worry if some text in the textboxes is cut off, as we still have the complete information in our database. Thank you!

Final step: Click submit !

Click submit so we have your answers in our database!

VERZENDEN

Verzend nooit wachtwoorden via Google Formulieren.

Deze content is niet gemaakt of goedgekeurd door Google. Misbruik rapporteren - Servicevoorwaarden

Google Formulieren
